# Age-specific alterations in gut microbiota-plasma metabolome networks in first-episode, drug-naïve major depressive disorder: adolescent versus adult patients

**DOI:** 10.3389/fmicb.2026.1922527

**Published:** 2026-09-03

**Authors:** Hui Mo, Dan Chai, LeTian Zhao, Jun Wang, Qiang Zhang, Hu Miaoyang, Ting Wang, WeiMin Lu, Changqing Wang

**Affiliations:** 1 Nanjing Hospital of Chinese Medicine Affiliated to Nanjing University of Chinese Medicine, Nanjing, China; 2 Department of Hygienic Analysis and Detection, Center for Global Health, Key Laboratory of Modern Toxicology, Ministry of Education, School of Public Health, Nanjing Medical University, Nanjing, China; 3 School of Automation and Intelligent Sensing, Shanghai Jiaotong University, Shanghai, China; 4 Jiangsu Province Hospital of Chinese Medicine Affiliated to Nanjing University of Chinese Medicine, Nanjing, China; 5 Nanjing University of Chinese Medicine, Nanjing, Jiangsu, China

**Keywords:** age heterogeneity, gut microbiota, major depressive disorder, metabolomics, microbiota-gut-brain axis

## Abstract

**Background:**

Major depressive disorder (MDD) exhibits pronounced age-related pathophysiological heterogeneity, yet the distinct signatures of the microbiota-gut-brain (MGB) axis between adolescent and adult patients remain inadequately characterized. This study aimed to elucidate age-specific MGB axis dysregulation by integrating gut microbiota profiling, plasma metabolomics, and their interactive networks in first-episode, drug-naïve MDD patients across these two developmental stages.

**Methods:**

We enrolled 62 first-episode, drug-naïve MDD patients (28 adolescents, 34 adults) alongside 43 age- and sex-matched healthy controls (22 adolescents, 21 adults). Fecal samples underwent 16S rRNA gene sequencing, while plasma was analyzed via untargeted metabolomics. Comprehensive bioinformatics and network analyses were applied to map microbial compositional shifts, metabolic perturbations, and microbe-metabolite crosstalks.

**Results:**

Integrated multi-omics analysis revealed distinct age-specific gut microbiota and plasma metabolome signatures in TMDD and AMDD compared with their age-matched controls. ① Microbiota: Beyond shared depletion of Firmicutes and butyrate-producing genera (Anaerobutyricum, Blautia), TMDD exhibited more extensive remodeling. Characterized by increased (Chao1 index, *p* < 0.05) Bifidobacterium loss and enrichment of Bacteroidetes, Gemmatimonadetes, and Planctomycetes. ② Metabolome: TMDD perturbations targeted neurodevelopmental and oxidative pathways, such as arginine/proline metabolism, D-amino acid metabolism, and glutathione metabolism. Whereas AMDD focused on energy dysfunction and excitotoxicity, involving the TCA cycle, purine metabolism, and alanine/aspartate/glutamate metabolism. ③ Interactome: In AMDD, positive correlations with protective (lipids) were attenuated, while pathological links with pro-inflammatory sphingolipids and polyamines strengthened. The interactome in AMDD remained stable.

**Conclusion:**

Our findings suggest that first-episode, drug-naïve MDD patients display distinct age-specific dysregulation of the microbiota-gut-plasma metabolome axis. The non-overlapping pathological signatures, a “development-nutrition-metabolic” disturbance pattern in adolescents versus an “energy metabolism-oxidative stress” pattern in adults, underscore the pronounced pathophysiological heterogeneity of MDD across the lifespan. These preliminary observations indicate that future diagnostic biomarkers and therapeutic interventions, particularly microbiota-targeted strategies, should be precisely tailored to the biological profiles of different age groups. However, given the exploratory nature of this subtyping, larger independent cohort studies are warranted to validate these findings before clinical translation.

## 1 Introduction

Major depressive disorder (MDD) remains a leading global cause of disability. Its prevalence continues to rise with a noticeable demographic shift toward younger populations, making Adolescents the fastest-growing affected group (Mokdad et al., 2016). Compared with Adult-onset MDD, Adolescent MDD often presents with atypical clinical features and a higher risk of suicide. Furthermore, its pathogenesis is intricately linked to pubertal neurodevelopment, hormonal fluctuations, and psychosocial transitions, necessitating the elucidation of age-specific mechanisms to enable precision intervention (Paus et al., 2008).

In recent years, the “microbiota-gut-brain (MGB) axis” has emerged as a critical framework for understanding MDD. The gut microbiota communicates bidirectionally with the central nervous system via immunological, metabolic, and neuroendocrine pathways (Cryan et al., 2019; Rutsch et al., 2020). Clinical studies have consistently reported gut dysbiosis in MDD, typically involving a loss of short-chain fatty acid (SCFA)-producing bacteria and an enrichment of pro-inflammatory taxa (Valles-Colomer et al., 2019). However, existing evidence is predominantly derived from Adult dominated cohorts, largely overlooking a fundamental biological reality: the gut microbiota is a dynamic ecosystem that undergoes programmed maturation from childhood through adolescence to adulthood (Yatsunenko et al., 2012). Consequently, age may be a decisive factor in the MGB axis mechanisms of MDD, making head-to-head comparative studies essential to unraveling the disorder’s heterogeneity.

MDD still lacks objective high-sensitivity biomarkers. Although single-omics approaches, such as neuroimaging and genomics, have made certain progress, they often suffer from insufficient sensitivity and specificity (Drysdale et al., 2017). Multi-omics integration, capable of capturing both the microbiome and host metabolome offers a promising strategy to identify robust biomarkers and age-specific pathological pathways (Hasin et al., 2017). To minimize confounding variables, this study focuses on first-episode, drug-naive (FEDN) patients. Providing a pristine pathological signal (Fava and Kendler, 2000).

**Figure 1:**
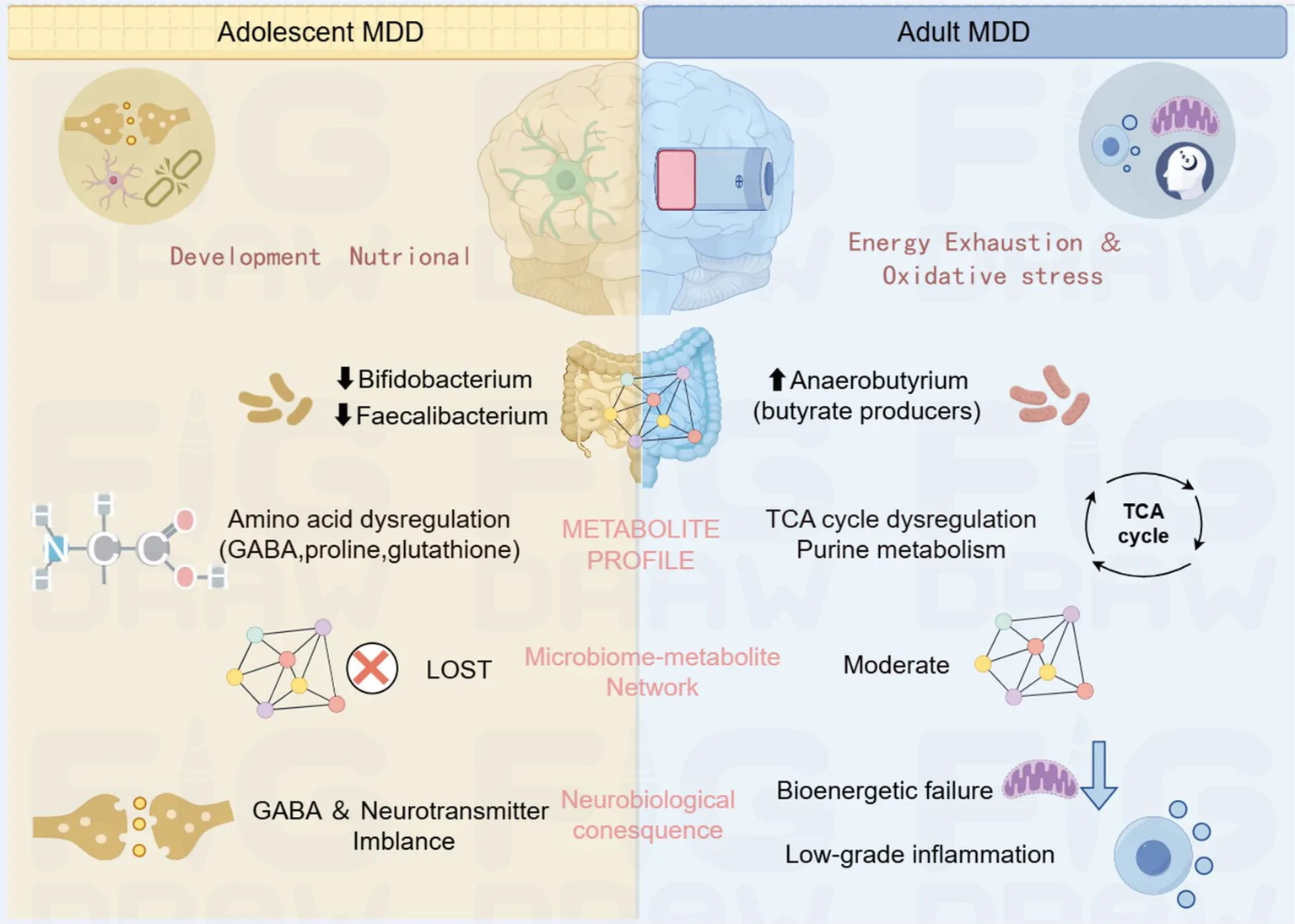
Age-specific gut-brain axis dysregulation mechanisms in first-episode, drug-naïve major depressive disorder: a comparative model of adolescent and adult patients. Adolescent MDD is characterized by a “development-nutrition-metabolic disturbance” pattern, including extensive gut dysbiosis, amino acid derivative metabolic disturbances, and disrupted protective microbe-metabolite correlations. Adult MDD exhibits an “energy metabolism-oxidative stress” pattern, characterized by loss of hub bacteria (e.g., *Blautia*), dysregulation of TCA cycle and purine metabolism, and collapse of the microbial-metabolite network.

To date, whether Adolescent and Adult MDD share a universal MGB axis signature or harbor divergent mechanisms remains unknown. Given that adolescence is a critical window of physiological remodeling, we propose a development-dependent model of MGB axis dysregulation (Figure 1). We hypothesize that Adolescent MDD is characterized by a “development-nutrition-metabolic” disturbance—involving extensive dysbiosis and amino acid metabolic shifts—whereas Adult MDD is driven by an “energy metabolism-oxidative stress” pattern, marked by the loss of hub taxa (e.g., Blautia) and the collapse of microbial-metabolite networks.

By integrating clinical assessments, gut microbiome profiling, and plasma untargeted metabolomics, this study aims to: (1) map and compare the multi-omics landscapes of FEDN Adolescent and Adult MDD; (2) identify age-specific biomarkers; and (3) construct correlation networks to elucidate differential MGB axis mechanisms. Our findings seek to provide empirical evidence for age-stratified diagnostic and therapeutic strategies in MDD.

## 2 Materials and methods

### 2.1 Study cohort and design

This single-center, observational cross-sectional design, supplemented by an exploratory longitudinal follow-up subgroup. A total of 62 first-episode, drug-naive patients (FEDN) with major depressive disorder (MDD) were recruited, comprising 28 Adolescents/Teenager (TMDD, aged 12–18 years) and 34 Adults (AMDD, aged 19–50 years). Simultaneously, 43 age-and sex-matched healthy controls (HC) were enrolled. Baseline blood samples were collected from all 105 participants. For fecal analysis, a subset of 93 participants (MDD: *n* = 57; HC: *n* = 36) provided baseline samples. All participants were recruited from the Nanjing Hospital of Chinese Medicine. The study protocol was approved by the Ethics Committee of Nanjing Hospital of Chinese Medicine Affiliated to Nanjing University of Chinese Medicine (No.: KY2024033). Written informed consent was obtained from all participants and their legal guardians prior to enrollment.

#### 2.1.1 Diagnosis and assessment

MDD diagnosis was established through face-to-face structured clinical interviews conducted by two senior psychiatrists using the Mini-International Neuropsychiatric Interview (MINI). Patients were required to meet the diagnostic criteria of the Diagnostic and Statistical Manual of Mental Disorders, Fifth Edition (DSM-5) and present a 24-item Hamilton Depression Rating Scale (HAMD-24) score of ≥20. HCs were recruited via community advertisements and underwent comprehensive screening to exclude current or lifetime history of psychiatric disorders. At baseline, demographic date (age, sex, and body mass index [BMI]) were recorded. A comprehensive psychometric battery was administered to evaluate clinical severity and psychological profiles, including the HAMD-24, 14-item Hamilton Anxiety Rating Scale (HAMA-14), Symptom Checklist-90 (SCL-90), Pittsburgh Sleep Quality Index (PSQI), Self-Rating Anxiety Scale (SAS), Self-Rating Depression Scale (SDS), Suicide Risk Score, and a the Connor-Davidson Resilience Scale (CD-RISC).

#### 2.1.2 Exclusion criteria

To ensure the purity of the pathological signal, participants were excluded based on the following criteria: (1) presence of other organic brain diseases; (2) co-occurrence of other psychiatric disorders; (3) history of chronic diseases or severe somatic illnesses; (4) current infectious diseases such as hepatitis B, HIV, or tuberculosis; (5) severe suicidal tendencies; (6) history of alcohol or other substance dependence or abuse; (7) family history of psychiatric disorders; (8) pregnant or lactating women, or participation in other clinical trials within the past 3 months; (9) acute gastrointestinal symptoms within the past 1 month; (10) use of antipsychotics, lithium salts, diuretics, or β-blockers within the past 2 weeks; and (11) use of antibiotics or probiotics within the past 3 months.

#### 2.1.3 Longitudinal subgroup

**Figure 2:**
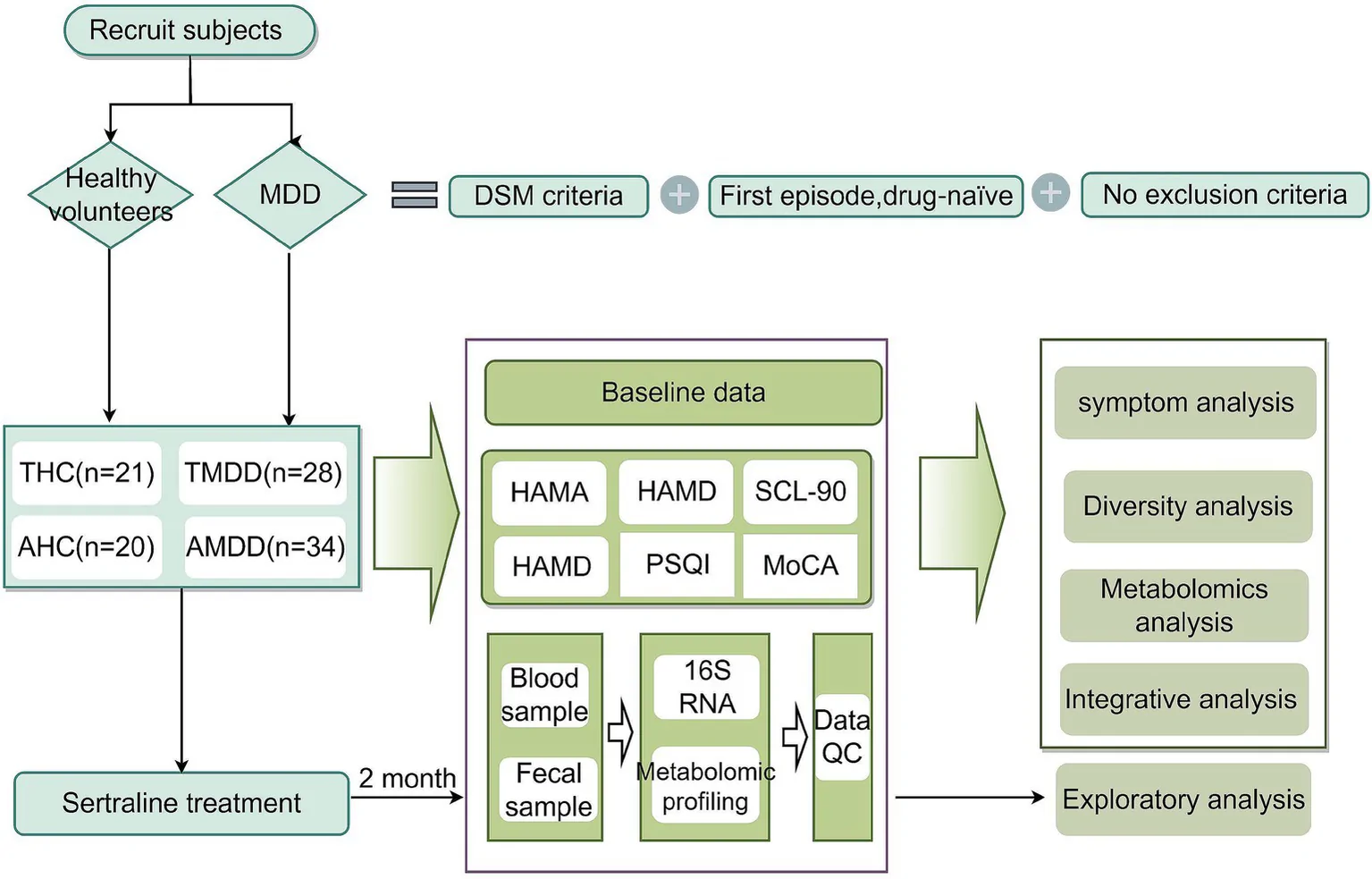
Study design and sample collection workflow. MDD, major depressive disorder; TMDD, teenager/adolescent MDD; A, adult MDD; THC, teenager/adolescent healthy controls; AHC, adult healthy controls.

Following the baseline assessment, a subgroup consisting of 19 patients (TMDD: *n* = 8; AMDD: *n* = 11) voluntarily received sertraline monotherapy (100 mg/day) and completed an 8-week follow-up. Blood and fecal samples were collected pre- and post-treatment for exploratory longitudinal analyses. Workflow is illustrated in Figure 2.

### 2.2 Plasma and fecal sample collection

All participants were instructed to maintain a standardized diet for 48 h and fast for at least 8 h prior to sampling. All venous blood samples were collected between 8:00 a.m. and 10:00 p.m. to minimize circadian rhythm effects using EDTA-coated anticoagulant tubes (Becton, Dickinson and Company, United States). The samples were centrifuged at 3000 rpm for 10 min at 4 °C. The resulting plasma was immediately aliquoted and stored at −80 °C until metabolomic analysis. Simultaneously, fresh fecal samples were collected. Processed following standard protocols, and stored at −80 °C for subsequent microbiome analysis.

### 2.3 Fecal microbiome analysis

Total genomic DNA was extracted from the fecal samples. The full length hypervariable regions of the bacterial 16S rRNA gene were amplified via PCR, and the resulting amplicons were sequenced on the PacBio Sequel II platform.

PacBio raw reads were processed using the SMRT Link Analysis software version 11.0 to obtain demultiplexed circular consensus sequence (CCS) reads with the following settings: minimum number of passes = 3, minimum predicted accuracy = 0.99. Raw reads were processed through SMRT Portal to filter sequences for length (>1,000 or <1800 bp) and quality. Sequences were further filtered by removing barcode and primer sequences with lima pipeline (Pacific Biosciences demultiplexing barcoded software, https://lima.how/).

OTUs were clustered with 98.65% (Edgar, 2018; Johnson et al., 2019; Gao et al., 2022) similarity cutoff using UPARSE (Edgar, 2013) (version 10, http://drive5.com/uparse/). The phylogenetic affiliation of each 16S rRNA gene sequence was analyzed by uclust algorithm (Edgar, 2010)^1^ against the Silva (SSU138.2) (Quast et al., 2013) 16S rRNA database^2^ using confidence threshold of 80% (Kim and Chun, 2014).

The rarefaction analysis based on Mothur v.1.21.1 (Wang N. et al., 2024) was conducted to reveal the diversity indices, including the Chao1, ACE, Simpson, and Shannon diversity indices (Schloss et al., 2009). The beta diversity analysis was performed using Euclidean distance matrix to principal component analysis (PCA) (Kim, 2014), and bray-curtis or UniFrac distance matrices to compare the results of the principal co-ordinates analysis (PCoA) (Luo et al., 2021) and non-metric multidimensional scaling (NMDS) with the vegan community ecology package, R-forge.^3^ Three complementary non-parametric multivariate statistical tests (Adonis) (Xiong et al., 2020; Algina and Keselman, 1999; Qiu et al., 2021) were performed using R vegan package to assess the statistically significant difference of bacterial communities diversity indices between samples.

Linear discriminant analysis Effect Size (LEfSe) and Wilcoxon rank-sum tests were employed to identify differentially abundant taxa. Only those with an LDA score >2.0 and an FDR-adjusted *q*-value <0.05 were reported.

Phylogenetic Investigation of Communities by Reconstruction of Unobserved States (PICRUSt2) (Wei et al., 2019)^4^ program based on the Kyoto Encyclopedia of Genes and Genomes (KEGG) database was used to predict the functional alteration of microbiota in different samples. The OTU data obtained were used to generate BIOM files formatted as input for PICRUSt2 with the make.biom script usable in the Mothur.

Spearman’s correlation analysis was employed for microorganisms and differential metabolites, with correlation coefficients ranging from −0.5 to 0.5 and *p* ≤ 0.08.

### 2.4 Plasma untargeted metabolomics analysis

Plasma untargeted metabolomic profiling was performed using ultra-performance liquid chromatography–tandem mass spectrometry (UPLC–MS/MS).

#### 2.4.1 Metabolite extraction

An aliquot of 100 μL plasma was mixed with 400 μL of pre-chilled 80% aqueous methanol. The mixture was vortexed and incubated on ice for 5 min, followed by centrifugation at 15000×g for 20 min at 4 °C. A specific volume of the supernatant was diluted with MS-grade water to a final methanol concentration of 53%. After a second centrifugation, the resulting supernatant was injected into the LC–MS system.

#### 2.4.2 Quality control (QC)

QC samples, prepared by pooling equal volumes from each experimental sample, were injected periodically to monitor instrumental stability and technical reproducibility.

#### 2.4.3 Chromatographic system

Separation was performed on a Thermo Vanquish UHPLC system equipped with a Hypersil Gold column (C18, 100 × 2.1 mm, 1.9 μm; Thermo Fisher, United States). The column temperature was maintained at 40 °C, with a flow rate of 0.2 mL/min. Mobile phase A consisted of 0.1% formic acid in water, and mobile phase B was methanol. The gradient elution program was: 0–1.5 min (2% B), 1.5–3.0 min (2–85% B), 3.0–10.0 min (85–100% B), and 10.1–12.0 min (2% B).

#### 2.4.4 Mass spectrometry

A Thermo Q Exactive HF-X mass spectrometer was used to acquire data in both positive and negative electrospray ionization (ESI) modes. The scan range was *m*/*z* 100–1,500. ESI parameters were: spray voltage 3.5 kV, sheath gas flow rate 35 psi, auxiliary gas flow rate 10 L/min, ion transfer tube temperature 320 °C, and auxiliary gas heater temperature 350 °C.

#### 2.4.5 Data processing and identification

The raw data files generated by UHPLC–MS/MS were processed using the Compound Discoverer 3.3 (CD3.3, ThermoFisher) to perform peak alignment, peak picking, and quantitation for each metabolite. The main parameters were set as follows: peak area was corrected with the first QC, actual mass tolerance, 5 ppm; signal intensity tolerance, 30%; and minimum intensity, et al. After that, peak intensities were normalized to the total spectral intensity. The normalized data was used to predict the molecular formula based on additive ions, molecular ion peaks and fragment ions. And then peaks were matched with the mzCloud,^5^ *mz*Vault and MassList database to obtain the accurate qualitative and relative quantitative results. Statistical analyses were performed using the statistical software R (R version R-3.4.3), Python (Python 2.7.6 version) and CentOS (CentOS release 6.6), When data were not normally distributed, standardize according to the formula: sample raw quantitation value/(The sum of sample metabolite quantitation value/The sum of QC1 sample metabolite quantitation value) to obtain relative peak areas; and compounds whose CVs of relative peak areas in QC samples were greater than 30% were removed, and finally the metabolites’ identification and relative quantification results were obtained.

These metabolites were annotated using the KEGG Database,^6^ HMDB Database,^7^ and LIPIDMAPS Database^8^ (Ping et al., 2015). Principal Component Analysis (PCA), Partial Least Squares-Discriminant Analysis (PLS-DA), and Orthogonal Partial Least Squares-Discriminant Analysis (OPLS-DA) were performed using the R package MetaboAnalystR.^9^ Univariate analysis (*t*-test) and one-way Analysis of Variance (one way-ANOVA) were employed to calculate statistical significance (*p*-value), respectively. For two-group comparisons, metabolites with OPLS-DA VIP ≥ 1, *p*-value ≤0.05, and fold change (FC) ≥ 2 or ≤0.5 were considered significantly differential metabolites; and *p*-values were adjusted for multiple testing using the Benjamini-Hochberg False Discovery Rate (FDR) method to generate *q*-values; for multi-group comparisons, metabolites with PLS-DA VIP ≥ 1 and *p*-value ≤0.05 were defined as significantly differential metabolites (Zhang et al., 2025). Using the R package ggplot2, visualizations including volcano plots, heatmaps, and lollipop plots of the significantly differential metabolites were generated based on their inter-group comparison statistics.

Based on the significantly differential metabolites identified from inter-group comparisons, the correlation and statistical significance among these metabolites were calculated using the base R function cor(). Heatmaps and chord diagrams were visualized using the R packages ggplot2 and circlize, respectively. To validate and screen metabolites with potential biomarker value, Receiver Operating Characteristic (ROC) analysis was performed for each significantly differential metabolite using the R package pROC,^10^ and random forest analysis was conducted for all significantly differential metabolites using the R package randomForest.^11^ The functions and metabolic pathways of these metabolites were explored using the KEGG Database, and metabolic pathway enrichment analysis was performed using the R package clusterProfiler.^12^ A metabolic pathway was considered a statistically significant enriched pathway when its *p*-value <0.05.

### 2.5 Statistical analysis

Statistical analyses were performed using SPSS (version 22.0) and GraphPad Prism (version 8.0). Continuous variables was expressed as mean ± standard deviation (SD), while categorical variables were presented as counts percentages. For normally distribution with homogeneity of variance, intergroup comparisons were conducted using Student’s *t*-tests or one-way analysis of variance (*ANOVA*; for multiple groups). For non-normally distributed data, Mann–Whitney *U* tests or Kruskal-Wallis *H* tests were employed. Categorical data were compared using the Chi-square test.

General linear regression models were utilized to examine the associations between microbial and metabolomic data. Receiver operating characteristic (ROC) curves were constructed to evaluate the predictive efficacy of specific biomarkers for clinical phenotypes, with performance quantified by the area under the curve (AUC). The stability of these predictive models was assessed using leave-one-out cross-validation. To account for multiple comparisons in high-dimensional omics datasets, the false discovery rate (FDR) was controlled using the Benjamini-Hochberg procedure. A corrected *q*-value <0.05 was considered statistically significant. For all other tests, a two-sided *p* < 0.05 was defined as the threshold for significance. Potential confounding variables, including age (within each group), sex, body mass index (BMI), were assessed for group balance using Chi-square tests (categorical variables) or Kruskal-Wallis tests (continuous variables). Given that groups were matched by age and sex, and no significant intergroup differences were observed in BMI (Table 1), these variables were not included as covariates in the primary analyses. However, we acknowledge that dietary habits and lifestyle factors—including physical activity levels, dietary fiber intake, and sleep patterns—were not systematically quantified in the present study. This represents a limitation, as these factors are known to influence gut microbiota composition and may differ between Adolescent and Adult participants (see Limitations). Co-occurrence networks between gut microbiota and plasma metabolites were constructed using Spearman correlation analysis. Correlations with *|r|* > 0.5 and FDR-adjusted *q*-value <0.05 were retained for network visualization. Procrustes analysis and Mantel tests were employed to assess the global concordance between microbiota and metabolite profiles.

**Table 1:** Clinical characteristics of the recruited major depressive disorder patients and healthy controls.

Characteristics	TMDD (teenager/adolescent MDD)	THC (teenager/adolescent healthy controls)	AMDD (adult MDD)	AHC (adult healthy controls)	*p*-value
Patients (*n*)	28	22	34	21	—
Gender (female/male)^a^	21/7	15/7	24/10	16/5	0.919
Age (years)					—
Range	12–18	12–18	19–50	19–50	
Mean ± SD^b^	15.00 ± 1.94	15.27 ± 1.88	28.18 ± 9.62	27.76 ± 8.92	
*p*-value	0.582		0.965		
BMI (kg/m^2^)					
Mean ± SD^b^	22.16 ± 6.75	21.90 ± 2.90	20.89 ± 4.08	22.71 ± 2.94	0.096
Duration of illness (months)^c^					
Mean ± SD^b^	13.9 ± 7.0	NA	13.9 ± 11.6	NA	0.319

Continuous variables are expressed as mean ± standard deviation (SD) or median (interquartile range, IQR), while categorical variables are presented as counts (percentage); BMI, body mass index; HAMD-24, 24-item Hamilton Depression Rating Scale; MDD: major depressive disorder; AMDD, adult MDD; TMDD, teenage/adolescent MDD.

a Analyzed using the Chi-square test.

b Analyzed using one-way analysis of variance (ANOVA) followed by Bonferroni post-hoc tests for intergroup comparisons.

c Analyzed using the Mann–Whitney *U* test.

## 3 Results

### 3.1 Demographic and clinical characteristics of the study population

No significant intergroup variations were observed among the four groups (TMDD, AMDD, THC, and AHC) in terms of sex distribution, body mass index (BMI),or other demographic variables (*p* > 0.05; Table 1).

No significant differences were observed between the two MDD groups regarding disease duration (13.9 ± 7.0 months for TMDD vs. 13.9 ± 11.6 months for AMDD, *p* = 0.319) or depression severity, including HAMD-24 scores (30.32 ± 7.94 vs. 29.74 ± 7.71, *p* = 0.745), SDS standardized scores (72.86 ± 10.95 vs. 72.65 ± 9.45, *p* = 0.538), and suicide scores (5.71 ± 3.67 vs. 5.12 ± 2.90, *p* = 0.477). These results indicate that both groups exhibited comparable baseline disease states (Table 2). However, a significant difference in sleep quality, as assessed by the PSQI, was identified between the groups. The total PSQI scores were significantly higher in the AMDD group than in the TMDD group (12.91 ± 3.83 vs.10.82 ± 2.36, *p* = 0.011), with AMDD patients demonstrating more pronounced impairments, particularly in sleep latency and subjective sleep quality.

**Table 2:** Comparison of clinical characteristics between TMDD and AMDD groups at baseline.

Characteristics	TMDD (teenager/adolescent MDD)	AMDD (adult MDD)	*p*-value
Patients (*n*)	28	34	
Gender (female/male)^a^	21/7	24/10	
Depression symptoms severity^b^			
HAMD-24 (mean ± SD)	30.32 ± 7.94	29.74 ± 7.71	0.745
Anxiety	6.68 ± 2.92	7.15 ± 2.36	0.488
Weight	0.61 ± 0.79	0.74 ± 0.86	0.547
Cognitive disturbance	7.61 ± 2.78	6.5 ± 3.11	0.149
Diurnal variation	1.14 ± 0.71	1.00 ± 0.70	0.427
Retardation	6.18 ± 2.78	6.06 ± 1.61	0.810
Sleep disturbance	3.32 ± 1.72	3.94 ± 1.60	0.147
Hopelessness	4.79 ± 2.13	4.35 ± 2.2	0.438
SDS standard score (mean ± SD)	72.86 ± 10.95	72.65 ± 9.45	0.538
Suicide score (mean ± SD)	5.71 ± 3.67	5.12 ± 2.90	0.477
Score of other associated symptoms^c^			
SAS (mean ± SD)	62.75 ± 9.31	62.24 ± 8.74	0.823
HAMA (mean ± SD)	23.89 ± 7.47	21.47 ± 9.31	0.270
**PSQI (mean ± SD)**	**10.82 ± 2.36**	**12.91 ± 3.83**	**0.011**
Subjective sleep quality	2 ± 0.72	2.29 ± 0.72	0.114
Sleep latency^†^	2.11 ± 0.96	2.53 ± 0.79	0.061
Sleep duration	1 ± 0.98	1 ± 1.01	0.173
Habitual sleep efficiency	1.5 ± 1.35	1.56 ± 1.44	0.87
Sleep disturbances	1.57 ± 0.69	2.59 ± 5.06	0.297
Use of sleeping medication	0.29 ± 0.76	0.68 ± 1.10	0.104
Daytime dysfunction	2.43 ± 0.84	2.66 ± 0.87	0.297
Other assessments			
Resilience scale	31.79 ± 15.01	35.70 ± 15.90	0.330
MoCA	27.39 ± 2.59	26.09 ± 3.05	0.078

Data are presented as mean ± standard deviation (SD). ^†^Indicates a trend toward statistical significance (*p* < 0.1).

a Differences in sex distribution were analyzed using the Chi-square test.

b Continuous variables were analyzed using either the independent samples *t*-test or the Mann–Whitney *U* test, depending on the data distribution.

c Values indicate a statistically significant difference between the two groups (*p* < 0.05).

Bold values indicate statistically significant differences.

### 3.2 Age-disease interaction effects on gut microbiota

Gut microbiota dysbiosis can influence depressive and anxiety-like behaviors (Foster and McVey Neufeld, 2013), and the composition of the gut microbiota varies significantly across different life stages (Odamaki et al., 2016). Therefore, investigating the differences in the gut microbiota of patients with MDD across different age groups is of great significance.

#### 3.2.1 Alpha and beta diversity

**Figure 3:**
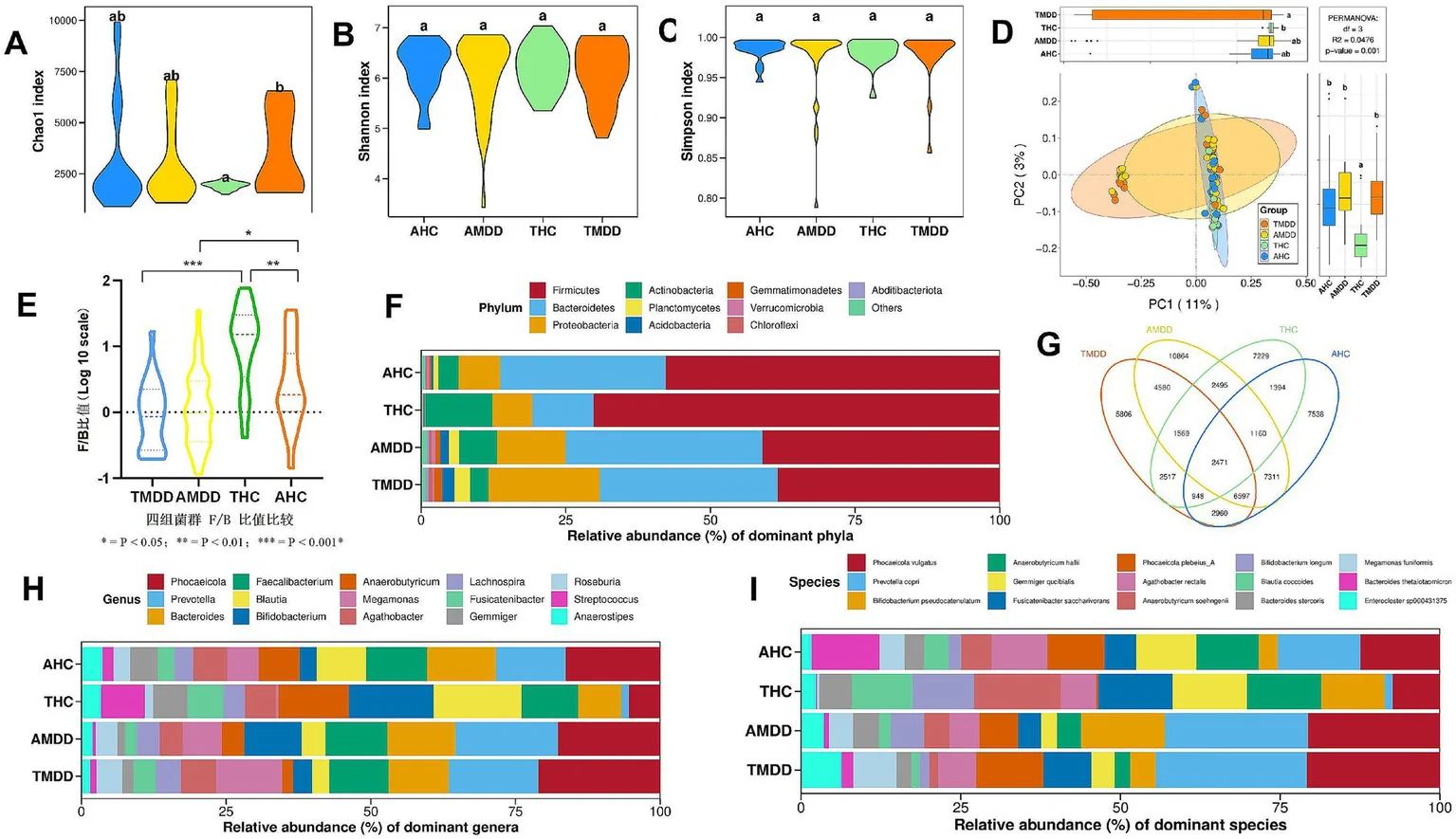
Comparison of gut microbiota diversity across THC, AHC, TMDD, and AMDD groups. **(A–C)** Alpha diversity indices (Chao1, Shannon, Simpson) shown as violin plots. **(D)** Beta diversity assessed by PCoA based on Bray-Curtis distance with PERMANOVA test results. **(E)** *F*/*B* ratio across four groups shown as boxplots (log10 scale); ***p < 0.001, **p < 0.01*p < 0.05 (Kruskal-Wallis with Dunn’s test). **(F)** Relative abundance of top 10 phyla across four groups. **(G)** Venn diagram of core OTUs across four groups. **(H)** Relative abundance of top 15 genera across four groups. **(I)** Relative abundance of top 15 species across four groups.

16S rRNA sequencing of fecal samples yielded a total of 76,159 operational taxonomic units (OTUs). The rarefaction curves indicated that the sequencing depth was sufficient for downstream analyses. Regarding alpha diversity, the Chao1 index was significantly higher in the TMDD group compared to the THC group (*p* < 0.05, Figure 3A). However, no significant differences were observed among the other groups (*p* > 0.05, Figure 3A). Furthermore, no significant differences in the Shannon and Simpson indices were detected among all groups (Figures 3B,C), which is consistent with previous findings (Liu et al., 2022).

Beta diversity analysis further revealed an age-specific segregation pattern of the microbiota. Principal coordinate analysis (PCoA) based on Bray-Curtis distances demonstrated a significant structural separation in the microbial community solely between the TMDD and THC groups (PERMANOVA, *p* = 0.001). Conversely, no significant differences were observed between the AMDD and AHC groups, nor between the two healthy control groups (Figure 3D).

#### 3.2.2 Differences in microbiota composition

Differences in microbial OTUs among the four groups were observed across the kingdom, phylum, class, order, family, genus, and species levels (Figure 3G). Overall, the taxa predominantly enriched in MDD patients belonged to the phylum *Bacteroidetes*, as well as the genera *Prevotella*, *Megamonas*. Conversely, the depleted taxa mainly belonged to the phyla *Firmicutes* and *Actinobacteria*, alongside the genera *Blautia*, *Anaerobutyricum*, *Gemmiger*, *Streptococcus*, and *Anaerostipes*.

At the phylum level, the gut microbiota was predominantly composed of *Firmicutes*, *Bacteroidetes*, *Proteobacteria*, *Actinobacteria*, *Planctomycetes*, *Acidobacteria*, and *Gemmatimonadetes*. At baseline, compared to the AHC group, the THC group exhibited an increased relative abundance of *Firmicutes* alongside decreased abundances of *Bacteroidetes*, *Planctomycetes*, and *Gemmatimonadetes*. Regarding disease-associated alterations, *Firmicutes* was depleted in both AMDD and TMDD patient groups relative to their respective controls. Furthermore, TMDD patients showed significant enrichments in *Bacteroidetes*, *Proteobacteria*, and *Gemmatimonadetes* compared to the THC group (Figure 3F). At the genus level, the dominant taxa included *Phocaeicola*, *Prevotella*, *Bacteroides*, *Faecalibacterium*, *Blautia*, *Bifidobacterium*, and *Anaerobutyricum*. *Anaerobutyricum* and *Blautia* were enriched in THC compared to AHC, but were notably depleted in both AMDD and TMDD groups. Interestingly, while most disease-associated alterations in Adolescents were consistent with those in Adults, they were substantially more pronounced. Specifically, the enrichment of *Phocaeicola*, *Megamonas*, as well as the depletion of *Prevotella* and *Anaerobutyricum*, were highly prominent in Adolescents. Notably, the genus Bifidobacterium exhibited an opposite trend between the two age groups, presenting a decrease in Adolescents but an increase in Adults (Figure 3H). At the species level, the THC group showed higher baseline abundances of *Anaerobutyricum hallii*, *Anaerobutyricum soehngenii*, *Fusicatenibacter saccharivorans*, and *Gemmiger gucibialls* than the AHC group. When comparing patients to healthy controls, TMDD exhibited a uniform depletion in all four of these species (*A. hallii*, *A. soehngenii*, *F. saccharivorans*, and *G. gucibialls*). In contrast, AMDD displayed a decrease in *F. saccharivorans* but an increase in *G. gucibialls* (Figure 3I).

Analysis of the Firmicutes-to-Bacteroidetes (F/B) ratio revealed that the F/B ratio in the THC group was significantly higher than those in the TMDD (*p* < 0.001) and AHC (*p* = 0.008) groups. Furthermore, the AHC group exhibited a significantly higher F/B ratio compared to the AMDD group (*p* = 0.045). However, no significant difference was observed between the TMDD and AMDD groups (*p* = 0.861) (Figure 3E).

**Figure 4:**
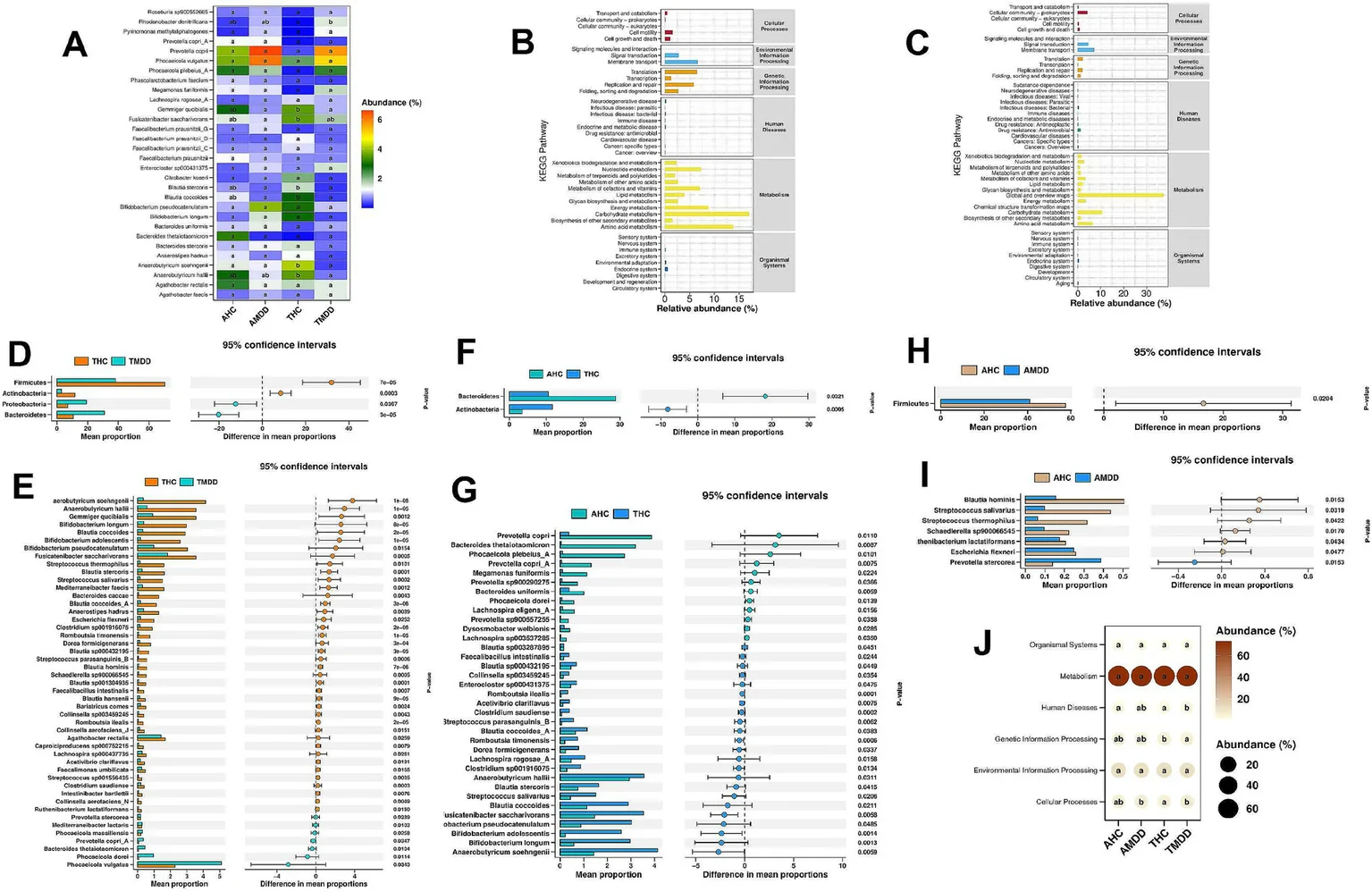
Taxonomic differences and functional predictions of gut microbiota. **(A)** Heatmap of top 30 species-level differential abundances across four groups; **(B)** KEGG pathway annotations (Levels 1 and 2) for THC vs. TMDD; **(C)** KEGG pathway annotations (Levels 1 and 2) for AHC vs. AMDD; **(D–I)** Wilcoxon rank-sum test results at phylum and species levels for pairwise comparisons: **(D)** THC vs. TMDD phylum, For the differential bacteria, all FDR-adjusted *q*-values <0.05; **(E)** THC vs. TMDD species; **(F)** AHC vs. THC phylum; For the differential bacteria, all FDR-adjusted *q*-values <0.05. **(G)** AHC vs. THC species; **(H)** AHC vs. AMDD phylum; For the differential bacteria, all FDR-adjusted *q*-values <0.05. **(I)** AHC vs. AMDD species; **(J)** Bubble plot of KEGG Level 1 pathway differences across four groups. For groups **(D–I)**, the FDR-adjusted *q*-values are provided in Appendix 2.

LEfSe and Wilcoxon tests further revealed age-dependent differences in the microbiota (Figures 4D–I). Age-related differences between the healthy control groups demonstrated that the THC group was enriched in taxa associated with carbohydrate metabolism, such as the genera *Prevotella* and *Bacteroides*. In contrast, the AHC group was dominated by butyrate-producing bacteria (e.g., the genus *Anaerobutyricum*) (Figures 4F–G), reflecting a more mature microbial homeostasis during adulthood.

Compared with their age-matched healthy controls, MDD patients generally exhibited a depletion in the phylum *Firmicutes*, alongside a decline in taxa such as *Blautia*, with concurrent reduction in Streptococcus. However, the TMDD group presented a markedly more pronounced microbial dysbiosis, involving the depletion of multiple butyrate-producing and anti-inflammatory bacteria (Figures 4D,E). The reduction in the genera *Bifidobacterium*, *Faecalibacterium*, *Collinsella*, *Agathobacter*, and *Lachnospira* was particularly notable. Notably, the AMDD group harbored a higher relative abundance of butyrate-producing bacteria (e.g. *Anaerobutyricum soehngenii)* and a lower abundance of potentially pathogenic bacteria (e.g. *Klebsiella pneumoniae*) (Figures 4H,I), suggesting a more stable and anti-inflammatory microbial profile.

#### 3.2.3 Functional prediction of the gut microbiota

PICRUSt2 was utilized to predict the functional profiles of the differentially abundant microbiota, revealing age-specific patterns of functional dysregulation. In the TMDD group, core functional pathways—such as transcription, translation, nucleotide metabolism, replication and repair, and nucleotide excision repair—were generally suppressed (Figures 4A, J). Additionally, the capacity for synthesizing essential nutrients (e.g., lysine biosynthesis, pantothenate and CoA biosynthesis) was decreased. Coupled with increased metabolic abnormalities, the core functions of the gut microbiota (particularly genetic information processing) were extensively inhibited (Figure 4B). Accompanied by severe impairments in nutrient synthesis, the TMDD group exhibited a phenotype characterized by “developmental impairment, nutritional deficiency, and metabolic abnormality.” It should be emphasized that these functional profiles were computationally predicted using PICRUSt2 based on 16S rRNA taxonomic data and do not represent direct measurements of microbial functional activity.

In contrast, the AMDD group was primarily characterized by the upregulation of pathways related to oxidative stress (glutathione metabolism) and lipid metabolism (PPAR signaling pathway), alongside a downregulation in RNA transport (Figure 4C). This profile suggests a phenotype driven by “oxidative stress and metabolic disorder.”

The altered bacterial genera possess significant functional implications. The genus Bacteroides plays a central role in host metabolism and immune regulation (Wexler, 2007), and its enrichment may be associated with the inflammatory state observed in MDD (Schiepers et al., 2005). The genera *Prevotella*, *Megamonas*, and *Roseburia* are closely linked to the production of short-chain fatty acids (SCFAs), particularly propionate and butyrate. Furthermore, *Faecalibacterium*, *Agathobacter*, and *Anaerobutyricum* are major butyrate producers. Notably, *Faecalibacterium* is strictly associated with psychiatric disorders such as depression (Nikolova et al., 2021) and positively correlates with quality-of-life indicators (Valles-Colomer et al., 2019). The extensive depletion of these beneficial bacteria leads to a decline in the metabolic function of the gut microbiome, a phenomenon that was particularly pronounced in the TMDD group.

#### 3.2.4 Microbiota-symptom associations

**Figure 5:**
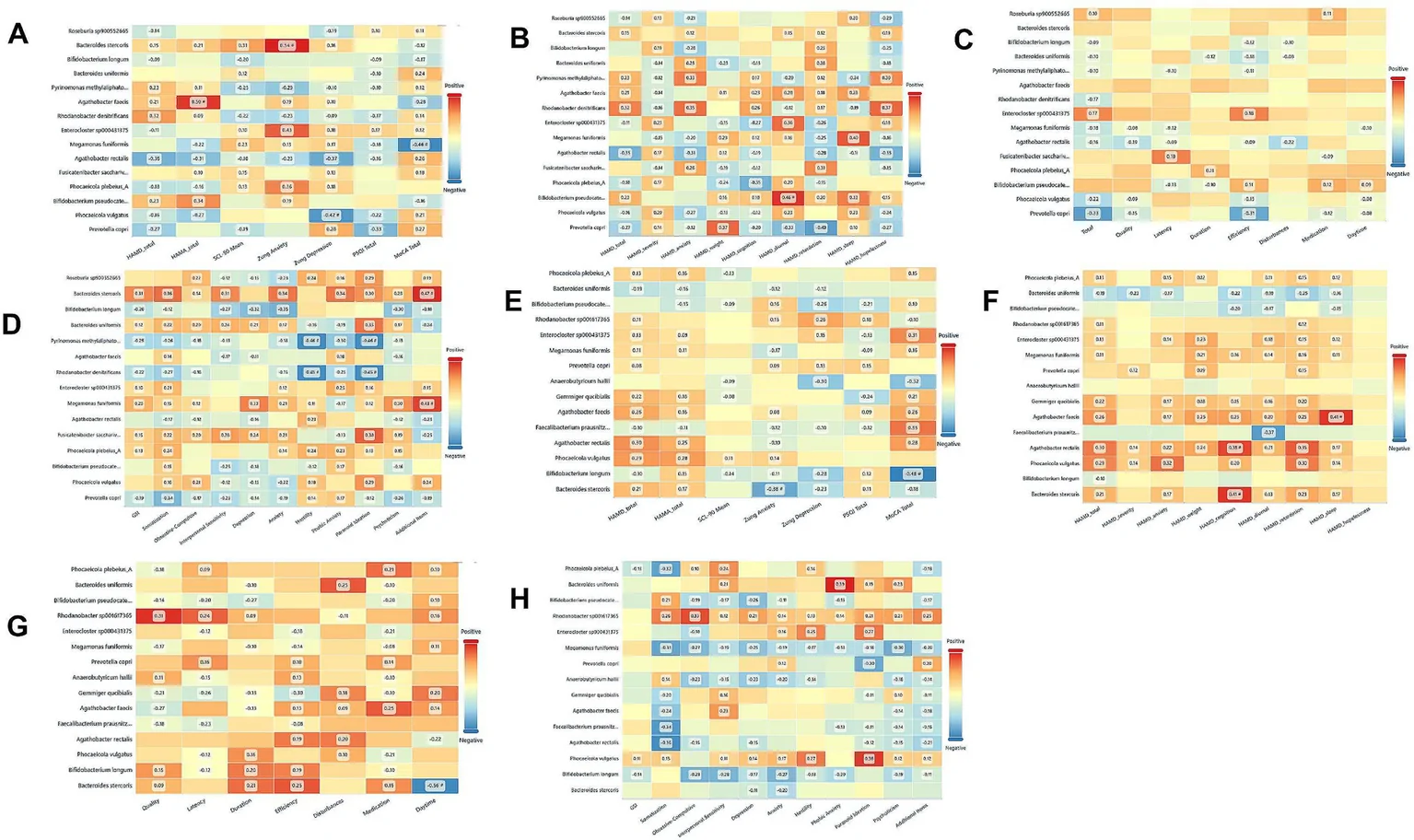
Correlation heatmap between gut microbiota (species level) and clinical symptom scores **(A–D)** Correlations in the TMDD vs. THC groups: **(A)** overall correlations; **(B)** HAMD subscales; **(C)** PSQI subscales; **(D)** SCL-90 subscales **(E–H)** Correlations in the AMDD vs. AHC groups: **(E)** overall correlations; **(F)** HAMD subscales; **(G)** PSQI subscales; **(H)** SCL-90 subscales. Color indicates the correlation coefficient *r* (red: positive, blue: negative). Only correlations with *p* < 0.05 are shown. ^#^FDR *q* < 0.05. For groups **(A–H)**, the FDR-adjusted *q*-values are provided in Appendix 2.

In the TMDD group, extensive associations were observed between the gut microbiota (at the species level) and clinical scale scores. These were predominantly characterized by positive correlations, and several of these associations survived FDR correction (Figure 5A). In contrast, these correlations were generally non-significant in the AMDD group, with no relationships surviving multiple testing corrections at the same threshold (Figure 5E).

*Specific associations in the TMDD group*: *Prevotella copri_A* exhibited significant negative correlations with multiple SCL-90 factors (somatization, obsessive-compulsive, depression, anxiety, and psychoticism; *r* ≈ −0.43 to −0.56) (Figure 5D). As the species with the most extensive associations, it potentially exerts a protective effect. Regarding the HAMD subfactors, cognitive impairment was negatively correlated with *P. copri_A* (*r* = −0.406, *q* = 0.045), and diurnal variation showed a positive correlation with *Bifidobacterium pseudocatenulatum* (*r* = 0.456, *q* = 0.0245) and a negative correlation with *P. copri_A* (*r* = −0.481, *q* = 0.0245) (Figure 5B). For the PSQI, sleep disturbances showed a negative correlation trend with *Agathobacter rectalis* (*r* = −0.220, *q* = 0.425), while sleep quality exhibited a negative correlation trend with *Gemmiger qucibialls* (*r* = −0.210, *q* = 0.241) and *Agathobacter faecis* (*r* = −0.266, *q* = 0.134). Daytime dysfunction was negatively correlated with *Prevotella copri* (*r* = −0.334, *q* = 0.215) and *Agathobacter rectalis* (*r* = −0.219, *q* = 0.425). (Figure 5C).

*Characteristics of the AMDD group*: Correlations in the AMDD group were generally weaker, and the association between somatization and *Agathobacter rectalis* was inconsistent across groups (Figures 5F–H). Specifically, in the AMDD group, *Agathobacter rectalis* showed a negative correlation trend with somatization (*r* = −0.3586, *q* = 0.055), while in the TMDD group, the correlation trend was positive (*r* = 0.1605, *q* = 0.490). Overall, the microbiota-symptom associations were far more pronounced in the TMDD group, highlighting the heterogeneity and distinct clinical mappings of gut microbiota characteristics in Major Depressive Disorder across different life stages.

### 3.3 Age-disease interaction effects on the plasma metabolome

#### 3.3.1 Global metabolic profiling and differential metabolites

**Figure 6:**
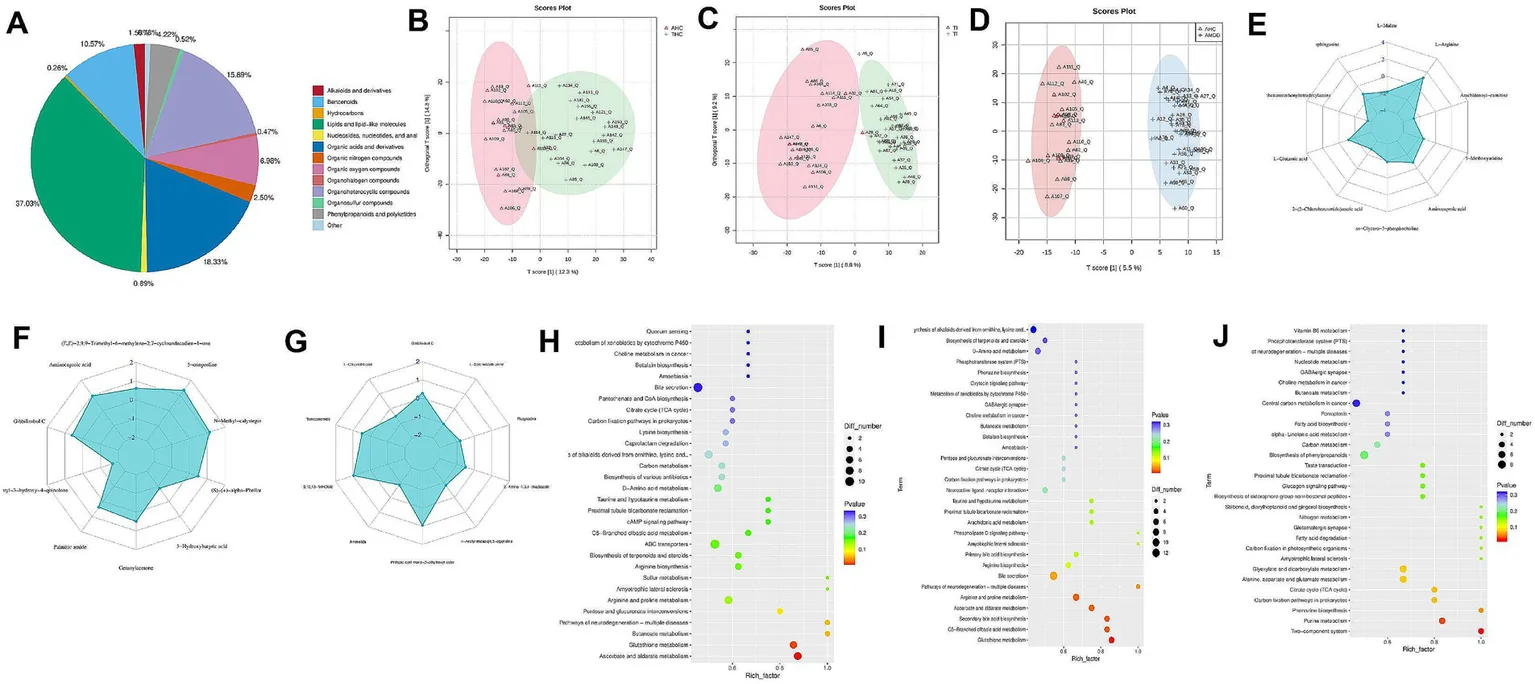
Plasma metabolic profiles and pathway alterations in adolescent and adult MDD patients compared to age-matched healthy controls **(A)** Pie chart showing the classification of detected metabolites (based on HMDB superclass); **(B–D)** OPLS-DA score plots **(B)** AHC vs. THC (*Q*^2^ = 0.411, *p* < 0.001); **(C)** THC vs. TMDD (*Q*^2^ = 0.507, *p* < 0.001); **(D)** AHC vs. AMDD (*Q*^2^ = 0.635, *p* < 0.001); **(E–G)** Radar charts of the top 10 differentially abundant metabolites **(E)** AHC vs. THC; **(F)** THC vs. TMDD; **(G)** AHC vs. AMDD; **(H–J)** KEGG pathway enrichment analysis bubble plots **(H)** AHC vs. THC; **(I)** THC vs. TMDD; **(J)** AHC vs. AMDD. All FDR values for the pathways in panels **(H–J)** were >0.05, and the results were not significant (details in Appendix 2).

A total of 1,920 metabolites were identified using Ultra-Performance Liquid Chromatography -Mass Spectrometry (UPLC-MS) (Figure 6A). OPLS-DA model analysis demonstrated a distinct separation of the metabolic profiles among the four groups (Figures 6B–D).

Based on the screening criteria (VIP > 1.5 and FDR-adjusted *q* < 0.2), we identified several differential metabolites across groups (Figures 6E–G):

(1) Metabolites enriched in the THC group: Included L-glutamic acid (an excitatory neurotransmitter), L-arginine, and arachidonoyl-carnitine (an intermediate marker of mitochondrial energy metabolism) (Figure 6E).
(2) Metabolites elevated in the TMDD group: Included 5-oxoproline (a glutathione precursor), Lyso-PAF C-16/C-18 (pro-inflammatory mediators), and aminocaproic acid (an amino acid derivative) (Figure 6F).
(3) Metabolic alterations in the AMDD group: Included 9,10,13-triHOME (a dihydroxy fatty acid metabolite), L-citramalic acid (an organic acid involved in energy metabolism), and maltotriose (Figure 6G).

#### 3.3.2 Age-specific metabolic pathway enrichment

KEGG pathway enrichment analysis of the differential metabolites revealed age- and disease-specific patterns of metabolic dysregulation (Figures 6H–J).

The metabolic profile of healthy Adolescents (THC) was particularly active, with enrichment in pathways such as bile secretion, arginine and proline metabolism, and glutathione metabolism, reflecting high metabolic demands and a robust antioxidant state. In the TMDD group, these pathways were disrupted, coupled with the emergence of dysregulated D-amino acid metabolism and ABC transporters. This suggests that metabolic disturbances in TMDD result from a “multiple-hit” mechanism, neurotoxicity, oxidative stress, and gut barrier dysfunction, superimposed on an age-specific metabolic background. Conversely, metabolic abnormalities in AMDD were primarily concentrated in purine metabolism, the TCA cycle, and alanine, aspartate, and glutamate metabolism (Figures 6H–J), highlighting a metabolic signature centered on energy dysregulation and oxidative stress.

#### 3.3.3 Metabolite-symptom correlations

The correlations between differential metabolites and various clinical symptom scores were analyzed separately for the TMDD and AMDD groups.

In the TMDD group, several notable metabolite-symptom associations emerged. Most prominently, following false discovery rate (FDR) correction, palmitic amide demonstrated a significant positive correlation with PSQI duration (*r* = 0.537, *q* = 0.024).

**Figure 7:**
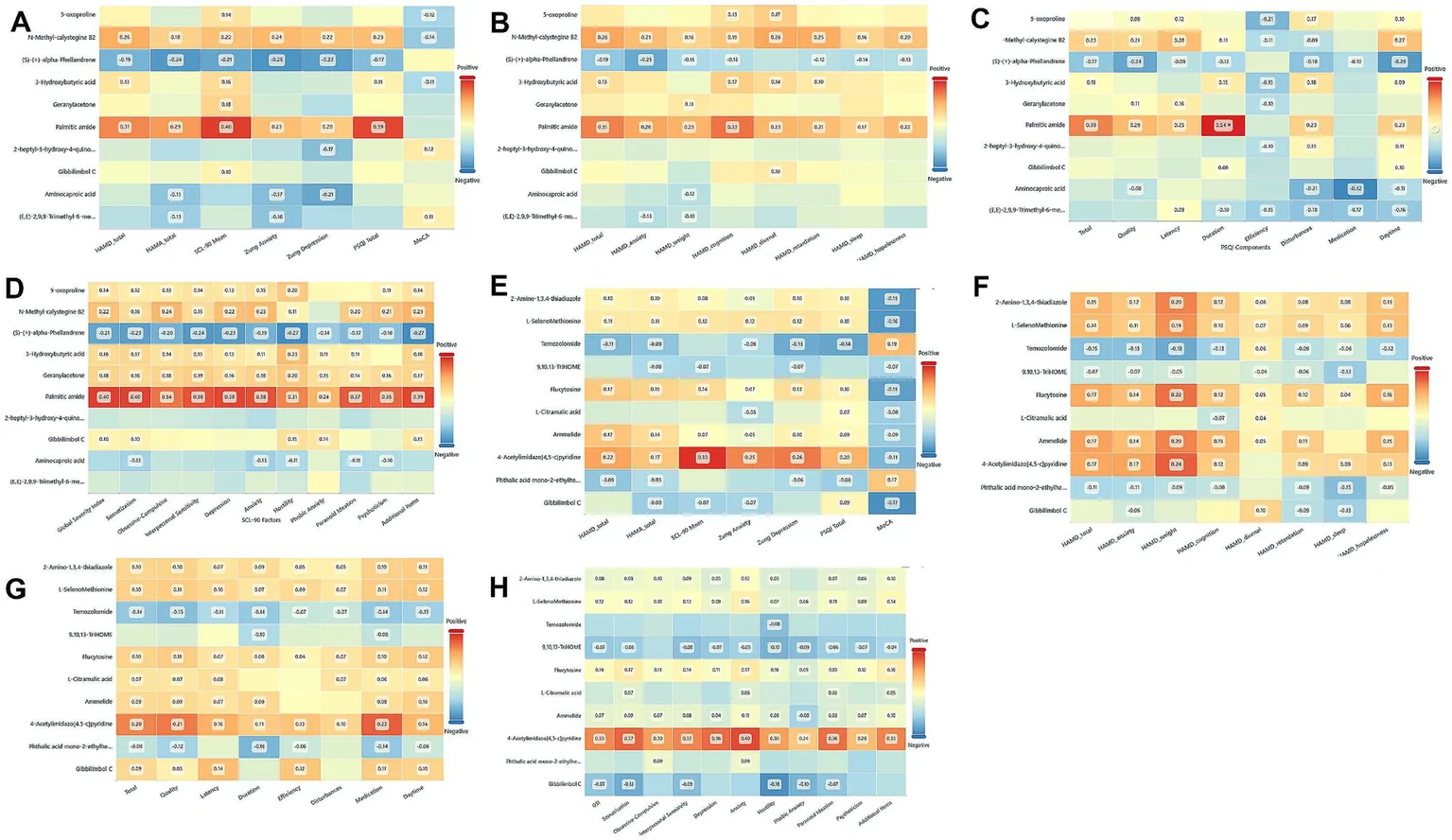
Correlations between plasma metabolites and clinical symptom scores. **(A–D)** Correlations in the TMDD vs. THC groups: **(A)** Overall correlations; **(B)** HAMD subscales; **(C)** PSQI subscales; **(D)** SCL-90 subscales. **(E–H)** Correlations in the AMDD vs. AHC groups: **(E)** Overall correlations; **(F)** HAMD subscales; **(G)** PSQI subscales; **(H)** SCL-90 subscales. Color and numbers indicate the correlation coefficient *r* (red: positive, blue: negative). Only correlations with nominal *p* < 0.05 are shown. GSI, Global Severity Index. FDR *q* < 0.05 (Across panels **(A–H)**, only Palmitic amide vs. PSQI duration showed an FDR *q* < 0.05). FDR-adjusted *q*-values for groups **(A–H)** are listed in Appendix 2.

It also exhibited marginally significant positive correlations with the SCL-90 Global Severity Index (GSI) (*r* = 0.404, *q* = 0.104) and its Somatization subscale (*r* = 0.399, *q* = 0.110). Beyond these specific metrics, palmitic amide displayed a consistent positive correlational trend across multiple comprehensive clinical scales, including the overall SCL-90 (*r* = 0.404), PSQI total score (*r* = 0.392), HAMD total score (*r* = 0.312), and HAMA total score (*r* = 0.286). These findings highlight its potential involvement in shared pathological mechanisms underlying emotional dysregulation and sleep disturbances. Furthermore, other metabolites demonstrated observable, albeit non-significant, trends. For instance, N-Methyl-calystegine B2 showed a positive trend with the total HAMD score (*r* = 0.261) and specific subscales such as diurnal variation (*r* = 0.256) and retardation (*r* = 0.249). Conversely, (S)-(+)-alpha-Phellandrene exhibited a negative correlational trend with anxiety metrics, notably the total HAMA score (*r* = −0.242) and the Zung Anxiety scale (*r* = −0.248) (Figures 7A–D).

In contrast, no associations in the AMDD group remained significant after adjusting for multiple comparisons (*q* < 0.05). This suggests that metabolite-symptom correlations in this cohort are either inherently weaker or constrained by the current sample size. Nevertheless, certain correlational trends were still discernible. Specifically, 4-Acetylimidazo[4,5-c]pyridine showed positive associations across several SCL-90 dimensions—including Anxiety (*r* = 0.397, *q* = 0.087), Somatization (*r* = 0.368, *q* = 0.104), and Depression (*r* = 0.362, *q* = 0.121), though none crossed the strict threshold for statistical significance. Similarly, Temozolomide displayed negative correlational trends with the HAMD weight factor (*r* = −0.181) and PSQI total score (*r* = −0.137); however, these associations also failed to achieve significance following FDR correction (Figures 7E–H).

#### 3.3.4 Age-specific diagnostic biomarkers

To evaluate the diagnostic potential of the identified metabolites, we constructed age-specific Random Forest models based on differential metabolites (VIP > 1.5, *p* < 0.05) and employed leave-one-out cross-validation to assess model performance. It should be noted that no independent external validation cohort was available in the present study; therefore, the diagnostic performance metrics reported here should be interpreted as internal estimates only, and the clinical utility of these models requires confirmation in future externally validated studies.

**Figure 8:**
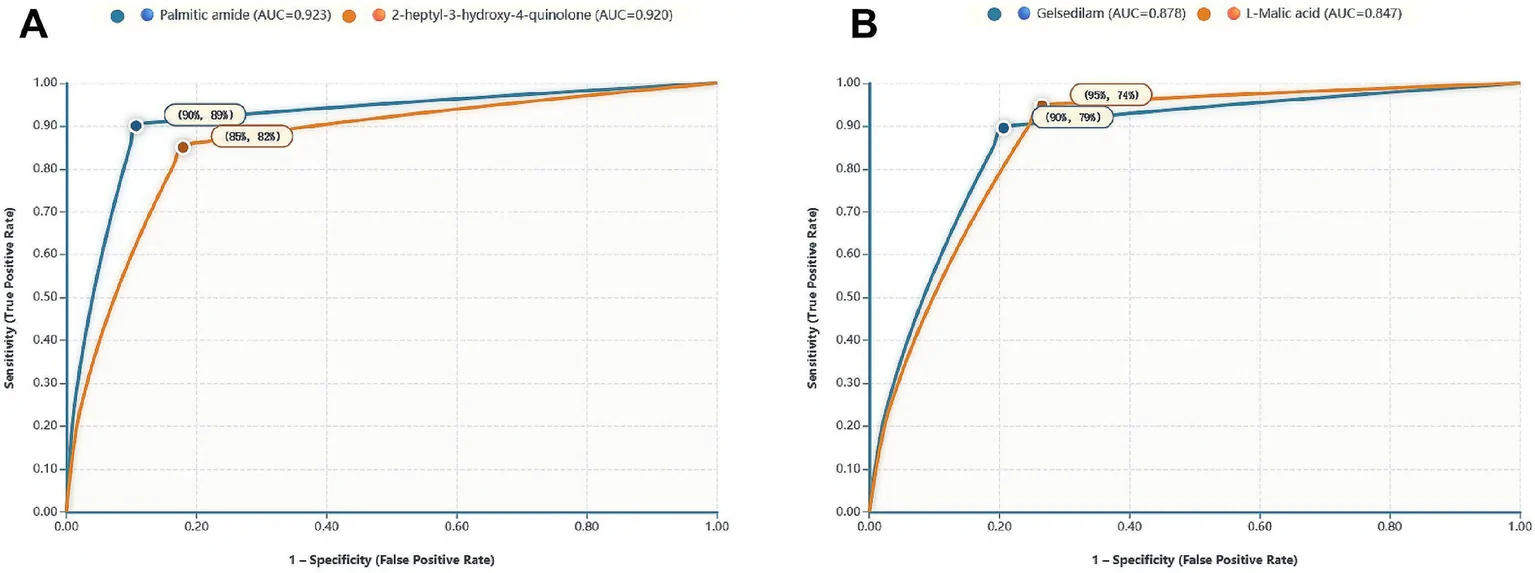
ROC analysis of age-specific metabolic biomarkers for MDD diagnosis. **(A)** High-performance metabolites in TMDD vs. THC (AUC > 0.90); **(B)** Key metabolites in AMDD vs. AHC (AUC < 0.89). ROC curves were evaluated using leave-one-out cross-validation; shaded areas represent 95% confidence intervals.

The TMDD vs. THC model demonstrated promising internal diagnostic accuracy (AUC = 0.94, 95% CI: 0.88–0.99). Furthermore, several metabolites exhibited outstanding univariate diagnostic power, such as palmitic amide (AUC = 0.923) and 2-heptyl-3-hydroxy-4-quinolone (AUC = 0.920) (Figure 8A). These findings suggest that Adolescent depression is characterized by intense and concentrated metabolic dysregulation signals.

In contrast, while the AMDD vs. AHC model showed good performance, its overall efficacy was slightly lower (AUC = 0.88, 95% CI: 0.81–0.95). The univariate diagnostic power of its key metabolites—such as gelsedilam (AUC = 0.878) and L-malic acid (AUC = 0.847)—was generally weaker (AUC < 0.89) (Figure 8B). Moreover, these metabolites were predominantly enriched in basal energy and nucleotide metabolism pathways. These results indicate that Adult MDD presents a more diffuse and heterogeneous pattern of metabolic imbalance compared to TMDD. However, given the absence of external validation, these AUC values likely represent optimistic estimates subject to overfitting.

### 3.4 Microbiota-metabolite correlation network: age-specific remodeling of the gut-brain axis

**Figure 9:**
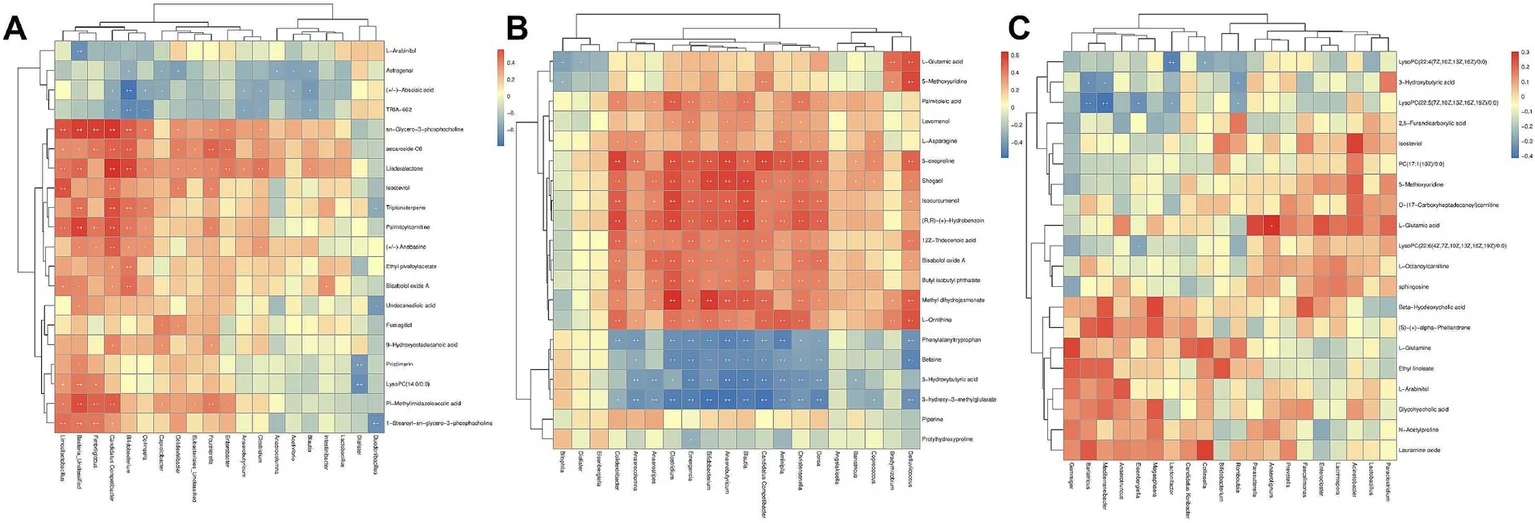
Age-specific microbial–metabolite correlation networks in health and depression. **(A)** Correlation heatmap between top 20 metabolites and gut microbiota in THC vs. AHC (Mantel_*p* = 0.341, Proc_*p* = 0.08); **(B)** Correlation heatmap in THC vs. TMDD (Mantel_*p* = 0.555, Proc_*p* = 0.664); **(C)** Correlation heatmap in AHC vs. AMDD (Mantel_*p* = 0.182, Proc_*p* = 0.672). Only correlations with |*r*| 0.5 and nominal *p* < 0.05 are shown. Red indicates positive correlation, blue indicates negative correlation. The intensity of color represents the magnitude of the correlation coefficient.

To investigate the global synergy between the gut microbiota and plasma metabolites and their alterations under diseased states, we performed Procrustes analysis and Mantel tests (Figure 9).

Age-related differences in healthy controls: The global structure of the microbiota-metabolite profiles in the THC group exhibited no significant concordance. In contrast, the AHCS group showed a moderate trend toward congruence (Mantel test: *r* = 0.112, *p* = 0.182; Procrustes analysis: M2 = 0.843, *p* = 0.097), characterized by an amine/bile acid metabolic network centered on the genus *Blautia* (Figure 9A). This suggests a developmental transition in gut-brain axis function across the lifespan.

Network remodeling in the TMDD group: In the TMDD group, the global concordance between the microbiota and metabolites vanished. Specifically (Figure 9B), the positive correlations between probiotics (e.g. *Bifidobacterium* and *Limosilactobacillus*) and anti-inflammatory lipids (e.g., *palmitoylethanolamide*, PEA) were attenuated. Conversely, positive associations between the microbiota and pro-inflammatory sphingolipids (e.g., *sphingosine*) and stress-related polyamines (e.g., spermidine) were strengthened. Furthermore, butyrate-producing bacteria and probiotics showed negative correlations with metabolites involved in fatty acid oxidation and ketogenesis. These findings indicate a pathological shift in the gut-brain axis of Adolescent MDD patients, transitioning from protective energy homeostasis toward neuroinflammation.

Network disintegration in the AMDD group: The AMDD group exhibited a distinct pattern of disruption (Figure 9C). Although the global synergy was not significant (Mantel test: *r* = 0.106, *p* = 0.201; Procrustes analysis: *p* = 0.672), its core feature was the complete disintegration of the *Blautia-*centered amine/bile acid metabolic network. Notably, the intense inflammatory-related rewiring observed in Adolescent MDD was absent in this group. This suggests that the gut-brain axis pathology in Adult MDD is more indicative of a systemic decline in core regulatory functions.

### 3.5 Exploratory analysis: age-specific responses to sertraline intervention

In a subgroup of 19 patients who completed the 8-week sertraline treatment, significant improvements from baseline were observed in both TMDD and AMDD groups regarding HAMD-24 and HAMA scores. However, no significant improvements were found in psychological resilience or MoCA scores. Although sleep quality appeared to improve more substantially in the Adult group than in the Adolescent group, these inter-group comparisons should be considered preliminary and interpreted with caution (Appendix 1 for details) due to the limited sample size (*n* = 19), which may lead to potential bias.

Both microbial and metabolic profiling revealed distinct responses to sertraline between the AMDD and TMDD groups (Supplementary Figures S6–S8). Diversity analysis showed no significant differences in α- or β-diversity across the four groups; however, microbial compositions were altered. A reduction in the phylum *Proteobacteria* was observed in both the TMDD-after and AMDD-after groups, while the phylum *Firmicutes* specifically increased in the AMDD-after group. Species enriched in the AMDD-after group (e.g. *Faecalibacterium prausnitzii* and *Roseburia intestinalis*) are primarily involved in the production of short-chain fatty acids (SCFAs), particularly butyrate. In contrast, the species that increased in the TMDD-after group (e.g. *Bacteroides thetaiotaomicron* and *Parabacteroides distasonis*) are predominantly associated with dietary polysaccharide fermentation and bile acid metabolism.

Metabolomic analysis further confirmed this age-specific divergence. In the TMDD-after group, differential metabolites (e.g., 13(S)-HODE and prednisolone) and the affected pathways were mainly involved in steroid hormone biosynthesis, ovarian steroidogenesis, aldosterone synthesis and secretion, and fatty acid metabolism (e.g., biosynthesis of unsaturated fatty acids and linoleic acid metabolism). Conversely, in the AMDD-after group, differential metabolites (e.g., 2-heptyl-3-hydroxy-4-quinolone) and pathway alterations were significantly directed toward amino acid and energy metabolism (including biosynthesis of amino acids, 2-oxocarboxylic acid metabolism, valine, leucine, and isoleucine biosynthesis, butanoate metabolism, and pyruvate metabolism) as well as lipid pathways (including sphingolipid metabolism and glycerophospholipid metabolism) (Appendix 1 for details).

## 4 Discussion

This study provides a systematic characterization of the clinical manifestations, gut microbiota, and plasma metabolic profiles, along with their integrated interaction networks, in patients with MDD across different developmental stages. Furthermore, we explored the longitudinal changes in these profiles following sertraline intervention. Our results suggest that dysregulation of amino acid derivative metabolism—specifically glutathione and D-amino acid metabolism may serve as a hallmark feature. Differentiating TMDD from AMDD. Additionally, we identified and internally validated a set of composite biomarkers capable of distinguishing TMDD and AMDD from their respective HC with high diagnostic precision. These findings lay a critical foundation for elucidating the role of age-related heterogeneity in the pathogenesis of MDD and offer potential pathways for the development of objective diagnostic tools.

Previous investigations into the gut microbiome of MDD have predominantly focused on Adult populations, often overlooking the distinct physiological and psychological developmental stages across the lifespan (Dabboussi et al., 2024; Zheng et al., 2020). Given that the composition and function of the gut microbiota undergo significant transitions with age (Odamaki et al., 2016), comparative studies between TMDD and AMDD remain insufficient. For instance, while prior research has reported an enrichment of *Bacteroidaceae* in MDD patients (Jiang et al., 2015), the age-specific nuances of these microbial shifts are rarely addressed. The innovation of the present study lies in its use of age as a primary stratification variable and its focus on first-episode, drug-naive MDD patients. By excluding mild cases to minimize clinical heterogeneity and integrating 16S rRNA sequencing with non-targeted metabolomics, we aimed to uncover intrinsic biological mechanisms unique to specific age groups and identify robust diagnostic markers.

### 4.1 Heterogeneity of clinical phenotypes: a focus on sleep disturbances

Our findings reveal that while Adolescent and Adult MDD patients share similarities in core symptoms, such as anxiety, cognitive impairment, and feelings of hopelessness, Adult patients exhibit more severe impairment in overall sleep quality, as indicated by higher total PSQI scores. However, considering the neurobiological development of sleep, this phenotypic divergence likely stems from distinct pathophysiological underpinnings.

Sleep is a central process for emotional regulation, and its disruption is intimately linked to depression. During adolescence, the sleep–wake system undergoes intensive remodeling, characterized by intrinsic circadian phase delays (Crowley et al., 2018), alterations in homeostatic sleep drive (Taylor et al., 2005), and a “structural-functional mismatch” resulting from the desynchronized development of the limbic system and the prefrontal cortex (Casey et al., 2008; Feinberg and Campbell, 2013). These factors render Adolescents more vulnerable to nocturnal emotional volatility and reward-seeking behaviors, which often lead to resistance against sleep pressure.

Consequently, sleep problems in TMDD should not be viewed simply as sleep deprivation; rather, they represent the convergence of intrinsic biological dysregulation—such as the microbial dysbiosis and metabolic abnormalities identified in this study—and the unique neurodevelopmental vulnerability of adolescence. This distinct sleep phenotype may be less responsive to standard pharmacotherapy and is deeply intertwined with affective symptoms (Li et al., 2018). In contrast, the severe sleep disturbances observed in AMDD more likely reflect compensatory functional dysregulation of fully matured sleep-regulatory circuits under chronic stress. This putative mechanistic difference may provide a preliminary clinical basis for distinguishing between the “Adolescent developmental-vulnerability” and “Adult metabolic-depletive” patterns of MDD, although this remains an exploratory hypothesis requiring longitudinal validation.

### 4.2 Age-specific gut dysbiosis and functional interpretation

We observed a reduction in the phylum *Firmicutes* in both TMDD and AMDD groups. However, the phylum *Bacteroide*tes increased in the TMDD group while remaining relatively stable in the AMDD group. At the genus level, the taxa enriched in MDD patients primarily included *Prevotella*, *Megamonas*, with microbial alterations being more extensive and pronounced in Adolescents than in Adults. Conversely, the depleted taxa mainly belonged to the genera *Blautia*, *Anaerobutyricum*, *Gemmiger*, *Streptococcus*, and *Anaerostipes* and *Roseburia*. Notably, the depletion of *Bifidobacterium*, *Faecalibacterium*, *Collinsella*, *Agathobacter*, and *Lachnospira* was particularly significant in TMDD patients. Interestingly, the genus *Bacteroides* showed a divergent pattern—increasing in TMDD but decreasing in AMDD, which contrasts slightly with some previous reports that found no significant changes in *Actinobacteria* (Yang et al., 2020; Zheng et al., 2016).

These microbial shifts may carry functional implications. The genus *Bacteroides* plays a pivotal role in host metabolism and immune regulation (Wexler, 2007), and its enrichment may be linked to the systemic inflammatory state of MDD (Schiepers et al., 2005). Furthermore, the synthesis of short-chain fatty acids (SCFAs) and the synthesis/degradation of neurotransmitters are closely associated with psychiatric disorders (Lai et al., 2021; Zhu et al., 2020).

At the genus level, our study identified a complex shift in SCFA-producing bacteria. Although certain genera such as *Prevotella* and *Megamonas* were enriched in MDD patients, the overall fecal butyrate concentration was significantly reduced. This clinical observation is likely driven by the widespread depletion of primary and more efficient butyrate producers, including *Streptococcus*, *Blautia*, *Anaerobutyricum*, and *Faecalibacterium*. Specifically, *Faecalibacterium* and *Anaerobutyricum* are recognized as core butyrate producers strongly associated with mental health (Nikolova et al., 2021) and various quality-of-life indicators (Valles-Colomer et al., 2019). Butyrate is essential for maintaining mucosal integrity and suppressing neuroinflammation via macrophage modulation. Therefore, the significant reduction of these beneficial taxa, which was more pronounced in the TMDD group, directly explains the observed decline in microbial metabolic capacity for butyrate synthesis and may exert a detrimental impact on Adolescent mental health.

A compelling finding in this study is that *Bifidobacterium* was depleted in TMDD but showed an increasing trend in AMDD. This may explain the inconsistent associations between *Bifidobacterium* and depression reported in various studies (Sanada et al., 2020). Given that *Bifidobacterium* abundance generally declines from infancy to adulthood (Odamaki et al., 2016) and is heavily influenced by diet, medication, and lifestyle in Adults, this age-related divergence suggests that the patterns and drivers of dysbiosis differ across the MDD lifespan. This paradoxical phenomenon indicates that the relationship between *Bifidobacterium* and depression is highly age-dependent, highlighting the need for future studies to incorporate dietary and other covariates to clarify the directionality of this association. This underscores the importance of controlling for dietary confounders in future age-stratified microbiome studies.

Several important confounding factors inherent to age-stratified microbiome research warrant discussion. The gut microbiota composition is profoundly influenced by dietary patterns, physical activity, sleep quality, and socioeconomic factors, all of which differ substantially between Adolescents and Adults in the general population. In the present study, while the four groups were matched for age and sex, systematic dietary assessment using a validated food frequency questionnaire (FFQ) was not conducted. It is well established that Adolescents typically consume diets higher in processed foods and lower in dietary fiber compared to Adults, while Adults may have higher alcohol consumption rates, all factors known to shape microbiota composition independently of disease status. Therefore, the age-group differences in microbiota composition reported here may partially reflect dietary and lifestyle differences rather than MDD-specific pathology. Future studies should incorporate detailed dietary profiling and lifestyle questionnaires to disentangle disease-related microbiota alterations from age-normative dietary influences.

### 4.3 Age-stratified host metabolic landscapes and their gut-microbiota associations

It is increasingly recognized that gut bacteria significantly modulate various host metabolic pathways. Inflammatory signals can be transmitted bi-directionally between the gut and the central nervous system via humoral, cellular immune, and neural routes (Agirman et al., 2021). Disruptions in microbial genes and host metabolites involved in carbohydrate and amino acid metabolism have been implicated in the pathogenesis of MDD (Zheng et al., 2016). Our metabolomic analysis further corroborates the fundamental biological divergence between TMDD and AMDD.

TMDD and AMDD exhibit distinct metabolic “epicenters” of dysregulation. TMDD patients manifest a metabolic phenotype characterized by disturbances in 5-oxoproline (a glutathione precursor) and D-amino acid metabolic pathways. This finding has profound pathophysiological implications, suggesting that the metabolic profile in TMDD may indicate reduced gut-derived GABA synthesis (Patterson et al., 2019) and GABA ergic system dysfunction (Prévot and Sibille, 2021). Since GABA metabolism shares precursors with glutathione synthesis, its disruption is associated with “multiple hits” encompassing neurotoxicity and oxidative stress (Maes et al., 2012). Consequently, TMDD may involve a combination of neurotransmitter imbalance, neurotoxicity, and oxidative damage. These features of “neurotrophic deprivation” and “neurochemical imbalance” occur during a critical window of synaptic pruning and circuit optimization, where neuronal networks possess high plasticity but extreme vulnerability (Paus et al., 2008), distinguishing Adolescent depression from its Adult counterpart.

Conversely, metabolic dysregulation in AMDD patients is centered on energy metabolism, characterized by the upregulation of the TCA cycle, purine metabolism, and alanine, aspartate, and glutamate metabolism. This may reflect a state of “metabolic-inflammatory depletion” driven by energy dyshomeostasis and oxidative stress. This state may stem from chronic energy metabolism impairment due to long-term occupational and life stressors in adulthood, as supported by previous literature (Manji et al., 2012; Picard and McEwen, 2018). However, these mechanistic interpretations remain speculative given the cross-sectional design of this study.

### 4.4 Integrated model: the “development-nutrition-metabolic disturbance” vs. “energy metabolism-oxidative stress” pattern hypothesis (exploratory)

Based on these exploratory cross-sectional findings, we tentatively propose a working hypothesis: the putative core pathological signatures of TMDD and AMDD appear fundamentally distinct and may be summarized as two preliminary patterns: the “development-nutrition-metabolic disturbance” pattern and the “energy metabolism-oxidative stress” pattern. We emphasize that these represent hypothesis-generating observations derived from a single cross-sectional cohort, and definitive characterization of these subtypes requires prospective longitudinal studies with functional validation.

Putative Adolescent “development-nutrition-metabolic disturbance” (TMDD): This pattern may be associated with a “failure of microbial scaffolding.” During critical neurodevelopmental windows (Paus et al., 2008), the gut microbiota has been proposed to act as a “scaffold” for brain maturation by providing immune, metabolic, and neurotrophic support (Heijtz et al., 2011). In TMDD, impairments in microbial pathways related to genetic information processing and the synthesis of essential plastic nutrients (e.g., pantothenate, lysine) may be associated with localized nutritional deficiencies in the brain (Magnúsdóttir et al., 2015). This vulnerability often originates from early-life adversity (the “first hit”), which serves as a mediator between MDD and suicidal ideation (Lee et al., 2025). Early-life adversity has been shown to persistently “program” the child’s gut microecology, increasing the risk for subsequent emotional disorders (Callaghan et al., 2020). When exposed to new psychosocial stressors during puberty (the “second hit”), this bi-directional dysregulation is acutely amplified (Foster et al., 2017), potentially contributing to the collapse of the “scaffolding” and manifesting as TMDD characterized by neurotrophic deprivation and neurotransmitter imbalance. However, whether these represent causative mechanisms or secondary consequences of MDD cannot be determined from the current cross-sectional design.

Putative Adult “energy metabolism-oxidative stress” (AMDD): This pattern may be associated with a “rigid dysregulation of metabolic-immune homeostasis.” Under chronic stress, prolonged activation of neuroendocrine systems (e.g., the HPA axis) may be associated with glucocorticoid resistance and persistent low-grade inflammation. This represents a “rigid” inflammatory state that has lost its regulatory flexibility (Miller and Raison, 2016; Wang K. et al., 2024). This state alters the gut microbiota-host relationship, depleting beneficial bacteria and allowing pro-inflammatory influx, which may exacerbate systemic metabolic-inflammatory imbalances (Tan, 2023). This may culminate in cellular energy failure and chronic inflammation (Steptoe et al., 2005), manifested as mitochondrial dysfunction and bioenergetic insufficiency (Klinedinst and Regenold, 2015). Previous studies have confirmed that Adult MDD is characterized by energy metabolism disturbances and inflammation-related metabolic shifts (Bharti et al., 2021), presenting as a “metabolic-inflammatory depletion” state. These interpretations are hypothesis-generating and require validation in longitudinal cohorts.

It is important to emphasize that these proposed patterns are derived from cross-sectional associations and represent working hypotheses, not validated subtypes. The modest sample size and lack of external replication preclude any claim of subtype classification. Future longitudinal and intervention studies are essential to test whether these patterns indeed reflect distinct pathophysiological trajectories.

### 4.5 The key hub of tryptophan metabolism

Tryptophan metabolism serves as a critical hub connecting the gut microbiota, systemic inflammation, and brain neurochemistry (Dantzer et al., 2008). In MDD, excessive pro-inflammatory cytokines overactivate indoleamine 2,3-dioxygenase (IDO) and tryptophan 2,3-dioxygenase (TDO), diverting tryptophan toward the kynurenine pathway at the expense of 5-HT production. This process synergizes with distinct pathological networks in TMDD and AMDD to exacerbate their respective core injuries. The mechanistic links described below are hypothetical and based on cross-sectional associations.

In TMDD, inflammatory hyperactivation of the kynurenine pathway synergizes with disturbances in arginine/proline, glutathione, and D-amino acid metabolism. Quinolinic acid, a neurotoxic downstream product of this pathway, is a potent agonist of NMDA receptors, potentially exacerbating glutamatergic excitotoxicity and inducing oxidative stress by depleting glutathione (Cheng et al., 2024; Schwarcz et al., 2012). Combined with the high plasticity of the developing brain, this may deliver “multiple hits” to maturing neural circuits, disrupting neurodevelopmental homeostasis.

In AMDD, chronic activation of the kynurenine pathway may form a vicious cycle with core cellular energy impairments. Continuous kynurenine pathway activation consumes substantial amounts of NAD+, a critical cellular energy substrate, thereby impairing mitochondrial function. This further intensifies oxidative stress and inflammation, creating a positive feedback loop that reactivates IDO (Oxenkrug, 2010). This “inflammation-energy depletion cycle,” in conjunction with glutamate metabolic disturbances, may drive progressive damage in the mature brain.

Based on these mechanisms, age-specific stratified strategies may warrant exploration: for TMDD, interventions should prioritize anti-inflammatory and antioxidant approaches to protect neurodevelopment; for AMDD, the focus should be on breaking the inflammation-energy depletion cycle to restore homeostasis (Bauer and Teixeira, 2019). These stratified strategies remain hypothetical and require validation through prospective interventional studies.

### 4.6 Strengths and limitations

This study possesses several notable strengths. First, based on a real-world clinical cohort, it represents the first integrated multi-omic analysis (encompassing clinical, microbial, and metabolic profiles) to systematically evaluate the biological divergence between TMDD and AMDD. By focusing exclusively on first-episode drug-naive patients, we effectively mitigated confounding factors such as disease duration and prior medication, allowing the identified microbial and metabolic signatures to reflect the nascent stages of the disorder. To our knowledge, this is the first study to prioritize age stratification in this context and provide a preliminary elucidation of the heterogeneity in MDD pathogenesis. However, as a cross-sectional study, these findings are hypothesis-generating and require prospective validation.

While providing novel insights into microbiota and metabolome in MDD interactions, our study has several limitations. First, the cross-sectional design precludes causal inference, rendering the reported associations purely observational. Prospective longitudinal cohorts tracking multi-omics dynamics from premorbid stages through treatment are required to establish directionality and mechanistic links.

Second, the modest, single-center sample size (TMDD: *n* = 28; AMDD: *n* = 34; THC: *n* = 22; AHC: *n* = 21) limits statistical power and generalizability. This is compounded by age stratification, which inherently reduces per-group sizes and increases type II error risks. Consequently, our Random Forest models, evaluated solely via internal leave-one-out cross-validation, require cautious interpretation. The small longitudinal cohort (*n* = 19) cannot substantiate robust biomarker claims; thus, the reported AUCs remain preliminary internal estimates pending validation in larger, independent cohorts.

Third, the absence of systematic dietary assessment represents an additional source of potential confounding, as differences in dietary fiber intake, physical activity, and alcohol consumption between Adolescent and Adult participants could not be formally adjusted for. Although participants were stratified into Adolescent and Adult groups, the gut microbiota undergoes continuous remodeling across the Adult lifespan, and finer age stratification, distinguishing, for instance, young Adults from middle-aged individuals, may reveal more granular microbial subtypes that the current grouping could not capture. Finally, our methodological scope is confined. PICRUSt2 derived functional profiles are computationally inferred from 16S rRNA amplicons and cannot substitute for direct metabolic measurements, necessitating future validation via shotgun metagenomics and metatranscriptomics. Furthermore, our exclusive focus on bacteriomes omits the gut virome and mycobiome, highlighting the need for comprehensive multi-kingdom profiling to fully capture the microbial landscape in MDD.

## Data Availability

The metabolomics dataset presented in this study has been deposited in the MetaboLights repository, accession number MTBLS15460. The raw sequence reads has been deposited in the NCBI BioProject database, accession number PRJNA1512643.

## Footnotes

1
https://github.com/topics/uclust

2
http://www.arb-silva.de

3
https://r-forge.r-project.org/

4
http://picrust.github.io/picrust/tutorials/genome_prediction.html

5
https://www.mzcloud.org/

6
https://www.genome.jp/kegg/pathway.html

7
https://hmdb.ca/metabolites

8
http://www.lipidmaps.org/

9
https://www.metaboanalyst.ca/docs/RTutorial.xhtml

10
https://xrobin.github.io/pROC/

11
https://www.stat.berkeley.edu/~breiman/RandomForests/

12
https://bioconductor.org/packages/release/bioc/html/clusterProfiler.html
