# Supplementary material for: Age-specific alterations in gut microbiota-plasma metabolome networks in first-episode, drug-naïve major depressive disorder: adolescent versus adult patients
Source: Front Microbiol. 2026 Sep 3;17:1922527. doi: 10.3389/fmicb.2026.1922527 (PMC13582463; doi:10.3389/fmicb.2026.1922527)
Supplement: Supplementary file 1 [file Data_sheet_1.DOCX]

**Appendix 1 : Comparative Analysis of Diversity and Metabolomics in TMDD Versus AMDD After Sertraline Treatment**

**1、Statistical analysis**

Categorical variables were analyzed using the Chi-square test. For continuous variables, independent samples t-tests were employed. Specifically, Levene’s test for equality of variances was first performed to assess the significance value (P). If the assumption of homogeneity of variance was met, the standard t-test was used; otherwise, the Satterthwaite-corrected (approximate) t-test was applied to ensure the robustness of the results.

Table S1：Changes in clinical scores before and after sertraline treatment in the exploratory subgroup

| **Characteristics** | **Teengers** | | | **Adult** | | |
| --- | --- | --- | --- | --- | --- | --- |
|  | TMDD before | H-TMDD after-treatment | P-value | AMDD before | H-AMDD after-treatment | P-value |
| Patients (n) | 8 | 8 |  | 11 | 11 |  |
| Depression symptoms severity ^c^ |  |  |  |  |  |  |
| HAMD-24 (Mean±SD) | 29.67±7.06 | 14.5±7.87 | 0.006 | 27.82±5.71 | 10.45±6.22 | 0.000 |
| Anxiety | 7.17±2.40 | 3.83±2.14 | 0.029 | 6.55±2.07 | 2.18±1.78 | 0.000 |
| Weight | 0.83±0.76 | 0.0±0.0 | 0.042 | 0.73±0.91 | 0.18±0.41 | 0.090 |
| Cognitive disturbance | 7.00±3.52 | 3.50±2.43 | 0.073 | 5.64±2.25 | 2.0±1.67 | 0.000 |
| Diurnal variation | 1.17±0.75 | 0.67±0.82 | 0.296 | 0.82±0.75 | 0.45±0.69 | 0.250 |
| Retardation | 5.67±2.16 | 3.67±1.97 | 0.124 | 6.09±1.22 | 2.55±1.75 | 0.000 |
| Sleep disturbance | 3.83±1.47 | 0.83±0.98 | 0.002 | 4.09±1.76 | 0.91±1.22 | 0.000 |
| Hopelessness | 4.00±0.89 | 2.33±1.63 | 0.053 | 3.91±1.97 | 1.82±1.47 | 0.011 |
| SDS standard score(Mean±SD) | 76.67 ± 6.22 | 61.17 ± 16.95 | 0.078 | 72.45±6.31 | 57.18±13.16 | 0.004 |
| Suicide score(Mean±SD) | 5.17±3.92 | 2.50±3.21 | 0.226 | 5.64±2.16 | 2.55±2.42 | 0.005 |
| Score of other associated symptoms^c^ |  |  |  |  |  |  |
| SAS(Mean±SD) | 63.00±6.66 | 54.0±11.40 | 0.126 | 58.82±8.13 | 48.64±13.92 | 0.049 |
| HAMA(Mean±SD) | 26.17±6.46 | 15.33±6.86 | 0.018 | 18.36±6.05 | 7.36±5.87 | 0.000 |
| PSQI(Mean±SD) | 10.5±1.05 | 8.00±3.90 | 0.182 | 12.91±4.23 | 9.27±2.45 | 0.025 |
| Subjective sleep quality | 2.0±0.63 | 1.17±0.75 | 0.065 | 2.45±0.69 | 1.45±0.52 | 0.001 |
| Sleep latency† | 2.17±0.41 | 1.0±0.89 | 0.016 | 2.73±0.47 | 1.55±0.52 | 0.000 |
| Sleep duration | 0.67±0.52 | 0.17±0.41 | 0.092 | 1.09±1.38 | 0.45±0.93 | 0.221 |
| Habitual sleep efficiency | 1.0±1.27 | 1.50±1.64 | 0.568 | 1.55±1.44 | 1.64±1.57 | 0.889 |
| Sleep disturbances | 1.67±0.52 | 1.17±0.41 | 0.092 | 1.36±0.81 | 1.0±0.45 | 0.207 |
| Use of sleeping medication | 0.33±0.82 | 0.83±1.17 | 0.411 | 1.18±1.33 | 1.00±1.10 | 0.73 |
| Daytime dysfunction | 2.67±0.52 | 2.17±0.98 | 0.304 | 2.55±0.93 | 2.18±1.08 | 0.408 |
| Other assessments |  |  |  |  |  |  |
| Resilience scale | 30.33±15.20 | 30.33±19.81 | 1.0 | 35.73±14.64 | 41.18±18.29 | 0.449 |
| MoCA | 26.33+2.34 | 27.67+2.42 | 0.355 | 25.0±2.79 | 25.64±2.50 | 0.58 |

Summary statistics: Distribution of variables across healthy controls (HCs) and patients with major depressive disorder (MDD). Continuous variables are expressed as mean ± standard deviation (SD), while categorical variables are presented as n (%).

Abbreviations: HAMD-24, 24-item Hamilton Depression Rating Scale; MDD, major depressive disorder.

a Analyzed using the Chi-square test.
b Analyzed using one-way analysis of variance (ANOVA) followed by Bonferroni post-hoc tests for intergroup comparisons.
c Analyzed using the Mann-Whitney U test.

**2. Microbial diversity and compositional shifts**

The comparison of microbial diversity across the four groups yielded the following results:
α-Diversity: No significant differences were observed in the Shannon, Simpson, and inverse Simpson indices among the groups (Fig. S1A–B).β-Diversity: Principal Coordinate Analysis (PCoA) indicated that the four groups did not exhibit distinct separation or significant differences in their primary components (Fig. S1D–E).

Despite the stability of overall diversity, sertraline intervention induced divergent shifts in microbial composition between the adolescent and adult groups. Analysis of the differential taxa revealed that both groups exhibited a reduction in the phylum Proteobacteria—a taxon typically associated with pro-inflammatory states—following treatment. In the AMDD group, a concurrent increase in the phylum Firmicutes was observed.

The specific species enriched in each group post-treatment pointed toward distinct functional pathways:

In the AMDD group: The enriched species were primarily short-chain fatty acid (SCFA) producers, particularly those involved in butyrate and acetate production. These included key butyrate-producing bacteria such as Faecalibacterium prausnitzii, Roseburia intestinalis, Agathobacter rectalis, and Fusicatenibacter saccharivorans(Fig. S1 I,K). The expansion of these taxa suggests that sertraline treatment in adults may strengthen intestinal mucosal integrity and enhance anti-inflammatory capacity.

In the TMDD group: Enrichment was observed in a different set of species, including Bacteroides thetaiotaomicron, Parabacteroides distasonis, and Mediterraneibacter lactaris. Most of these taxa are specialized in the fermentation of various dietary polysaccharides and the metabolism of primary bile acids. This suggests that the adolescent microbial response to sertraline possesses a unique functional emphasis, potentially favoring enhanced carbohydrate degradation and energy harvesting(Fig. S1H,J).

A B C

D E

F G

H I

J K

Figure S1. Alterations in gut microbiota diversity and composition across the four groups.

C) Comparisons of α-diversity among the four groups.

(D, E) Evaluation of β-diversity illustrating the structural variations of microbial communities among the four groups.

(F, G) Relative abundances of microbial taxa at the genus (F) and species (G) levels.

(H-I) Wilcoxon rank-sum test analysis of differentially abundant taxa at the phylum (H, I) and species

(J, K) levels. Pairwise comparisons demonstrate the differences before and after sertraline treatment in the TMDD (H, J) and AMDD (I, K) groups.

**3. Differential Metabolomic Profiles**

Our metabolomic analysis revealed significantly distinct systemic responses to sertraline intervention between adolescent and adult patients with major depressive disorder (MDD). In the adolescent group, although the OPLS-DA model exhibited a separation trend (Q2=0.508), it did not reach statistical significance (permutation test p=0.091)(Fig. S2A-B), which may be attributed to the relatively small sample size. Volcano plots identified a series of potential differential metabolites altered between the groups. Specifically, the differentially expressed metabolites in the TMDD (teenage MDD) group following sertraline treatment included 3(S)-HODE, prednisolone, palmitic amide, and N-hexanoylsphinganine (Fig. S2C) .Conversely, the differential metabolites in the AMDD (adult MDD) group treated with sertraline comprised 2-heptyl-3-hydroxy-4-quinolone, pramoxine, and andrachamine (Fig. S2D).

Regarding metabolic pathways, sertraline commonly altered several pathways in both groups, including glycerophospholipid metabolism, sphingolipid metabolism, and the serotonergic synapse pathway. However, the sertraline intervention in the TMDD group predominantly affected steroid hormone biosynthesis (e.g., steroid hormone biosynthesis, ovarian steroidogenesis, and aldosterone synthesis and secretion) and fatty acid metabolism (e.g., biosynthesis of unsaturated fatty acids and linoleic acid metabolism) (Fig.S2 E).

In contrast, the AMDD-sertraline group showed a bias towards microbial metabolism (such as quorum sensing and the biosynthesis of various antibiotics), amino acid and energy metabolism (including biosynthesis of amino acids, 2-oxocarboxylic acid metabolism, valine, leucine and isoleucine biosynthesis, butanoate metabolism, and pyruvate metabolism), as well as variations in lipid pathways (including sphingolipid metabolism and glycerophospholipid metabolism). Collectively, these findings suggest that the metabolic mechanisms underlying the antidepressant effects of sertraline may vary significantly across different age stages (Fig.S2 F).

A B

C D

E F

Figure S2 Metabolomic alterations following sertraline intervention in the TMDD and AMDD groups.

(A, B) Orthogonal partial least squares discriminant analysis (OPLS-DA) score plots comparing the metabolic profiles before and after sertraline treatment in the TMDD (A) and AMDD (B) groups.

(C, D) Differentially expressed metabolites induced by sertraline in the TMDD (C) and AMDD (D) groups.

(E, F) Metabolic pathway enrichment analysis of the differential metabolites in the TMDD (E) and AMDD (F) groups.
