# Supplementary material for: Age-specific alterations in gut microbiota-plasma metabolome networks in first-episode, drug-naïve major depressive disorder: adolescent versus adult patients
Source: Front Microbiol. 2026 Sep 3;17:1922527. doi: 10.3389/fmicb.2026.1922527 (PMC13582463; doi:10.3389/fmicb.2026.1922527)
Supplement: Supplementary file 2 [file Data_sheet_2.DOCX]

附录2 FDR矫正值

剩下 figure 4 多样性 、figure 9多样性和代谢的联合分析 的矫正值

Figure 4 Taxonomic differences and functional predictions of gut microbiota.(A)Heatmap of top 30 species-level differential abundances across four groups;(B) KEGG pathway annotations (Level 1 & 2) for THC vs. TMDD ;(B) KEGG pathway annotations (Level 1 & 2) for AHC vs. AMDD;(D-I) Wilcoxon rank-sum test results at phylum and species levels for pairwise comparisons: (D) THC vs. TMDD phylum; (E) THC vs. TMDD species; (F) AHC vs. THC phylum; (G) AHC vs. THC species;(H) AHC vs. AMDD phylum; (I) AHC vs. AMDD species;(J) Bubble plot of KEGG Level 1 pathway differences across four groups.

Figure 4-D THC vs. TMDD phylum Wilcoxon rank-sum test results at phylum and species levels for pairwise comparisons

| THC-TMDD 门水平 | p.value | fdr |
| --- | --- | --- |
| Bacteroidetes | 2.1205524201461e-05 | 0.000118810489728005 |
| Firmicutes | 2.97026224320014e-05 | 0.000118810489728005 |
| Actinobacteria | 0.000329689798579077 | 0.000879172796210873 |
| Acidobacteria | 0.0162584607152032 | 0.0325169214304064 |

Figure 4-E THC vs. TMDD species Wilcoxon rank-sum test results at phylum and species levels for pairwise comparisons

| THC-TMDD 种水平 | p.value | fdr |
| --- | --- | --- |
| Romboutsia timonensis | 2.25507748047973e-06 | 0.000104886793309725 |
| Romboutsia ilealis | 2.33081762910499e-06 | 0.000104886793309725 |
| Bifidobacterium adolescentis | 4.07690956698866e-06 | 0.00012230728700966 |
| Blautia sp000432195 | 1.6991001209096e-05 | 0.000343408314598097 |
| Blautia coccoides | 2.1205524201461e-05 | 0.000343408314598097 |
| Clostridium saudiense | 2.28938876398732e-05 | 0.000343408314598097 |
| Clostridium sp001916075 | 3.83148657011967e-05 | 0.000458452701744265 |
| Streptococcus salivarius | 4.24049068091508e-05 | 0.000458452701744265 |
| Blautia coccoides_A | 4.58452701744265e-05 | 0.000458452701744265 |
| Fusicatenibacter saccharivorans | 7.75673455504793e-05 | 0.000698106109954313 |
| Blautia hominis | 9.09652568029304e-05 | 0.000744261192023976 |
| Blautia hansenii | 0.000163788463828612 | 0.00109950783449917 |
| Intestinibacter bartlettii | 0.000163986035441913 | 0.00109950783449917 |
| Streptococcus thermophilus | 0.000171034552033204 | 0.00109950783449917 |
| Anaerobutyricum hallii | 0.000193465817057767 | 0.0011607949023466 |
| Bifidobacterium longum | 0.000228061045969077 | 0.00128284338357606 |
| Phocaeicola dorei | 0.00033620878782747 | 0.00177992887673366 |
| Blautia stercoris | 0.000360836143999225 | 0.00180418071999612 |
| Bacteroides thetaiotaomicron | 0.000528781542780682 | 0.00250475467632955 |
| Dorea formicigenerans | 0.000938743043779705 | 0.00422434369700867 |
| Mediterraneibacter faecis | 0.00112852818693738 | 0.00483654937258878 |
| Blautia sp001304935 | 0.00121171854246719 | 0.00495703040100215 |
| Anaerobutyricum soehngenii | 0.0015049778658187 | 0.00588904382276883 |
| Gemmiger qucibialis | 0.00185059841638841 | 0.00693419324528825 |
| Acetivibrio clariflavus | 0.00192616479035785 | 0.00693419324528825 |
| Escherichia flexneri | 0.00210825065546774 | 0.00729779073046526 |
| Phocaeicola massiliensis | 0.00260572843984322 | 0.00867068898731701 |
| Schaedlerella sp900066545 | 0.00277631845853262 | 0.00867068898731701 |
| Collinsella aerofaciens_J | 0.00279388867369104 | 0.00867068898731701 |
| Phocaeicola vulgatus | 0.00319662478814314 | 0.00958987436442942 |
| Lachnospira sp000436535 | 0.00334058733162272 | 0.00969847934987241 |
| Parabacteroides distasonis | 0.00413271371486648 | 0.011623257323062 |
| Prevotella stercorea | 0.00732752492482286 | 0.0199841588858805 |
| Lachnospira rogosae_A | 0.00763610723122748 | 0.0202132250238374 |
| Prevotella sp900557255 | 0.00892867286013875 | 0.0229594444974997 |
| Anaerotignum faecicola | 0.00945646827862792 | 0.0236411706965698 |
| Prevotella copri_A | 0.0109934440833086 | 0.0267408099323723 |
| Enterocloster sp000431375 | 0.0114110186022963 | 0.0270260966896492 |
| Bariatricus comes | 0.0117484557374976 | 0.0271118209326869 |
| Bacteroides uniformis | 0.0129231965217437 | 0.0290313418153976 |
| Lachnospira sp003537285 | 0.0132253890492367 | 0.0290313418153976 |
| Faecalimonas umbilicata | 0.0139789847002249 | 0.0299549672147677 |
| Caproiciproducens sp000752215 | 0.0166024093942681 | 0.0347492289647472 |
| Bifidobacterium pseudocatenulatum | 0.0185030500401258 | 0.0378471478093483 |
| Agathobacter rectalis | 0.0207137626021921 | 0.0405269268303758 |
| Prevotella copri | 0.0207137626021921 | 0.0405269268303758 |
| Prevotella sp900290275 | 0.0213980307308631 | 0.0409749524633548 |
| Bacteroides ovatus | 0.0246357914897539 | 0.0461921090432886 |
| Lachnospira sp000437735 | 0.0300658677522497 | 0.0552230224020912 |
| Anaerostipes hadrus | 0.0331763641383021 | 0.0597174554489438 |
| Faecalibacterium prausnitzii_G | 0.034031154561539 | 0.0600549786380099 |
| Bacteroides xylanisolvens | 0.0384343179247893 | 0.0665209348698277 |
| Parasutterella excrementihominis | 0.0448837414011942 | 0.0762176740774996 |
| Phascolarctobacterium faecium | 0.0469212464757295 | 0.0782020774595492 |
| Agathobacter faecis | 0.0489872867347993 | 0.0801610146569443 |

Figure 4-F AHC vs. THC phylum Wilcoxon rank-sum test results at phylum and species levels for pairwise comparisons

| THC-AHC 门水平 | p.value | fdr |
| --- | --- | --- |
| Actinobacteria | 0.00052609965556718 | 0.00368269758897026 |
| Bacteroidetes | 0.00217285971360547 | 0.00760500899761915 |

Figure 4-G AHC vs. THC species Wilcoxon rank-sum test results at phylum and species levels for pairwise comparisons

| THC-AHC Species | p.value | fdr |
| --- | --- | --- |
| Romboutsia ilealis | 0.000202545440163798 | 0.0106941995145471 |
| Clostridium saudiense | 0.000218248969684635 | 0.0106941995145471 |
| Romboutsia timonensis | 0.000691946259127184 | 0.0226035777981547 |
| Bifidobacterium adolescentis | 0.00149364785666936 | 0.0299853803393633 |
| Bifidobacterium longum | 0.00152986634384507 | 0.0299853803393633 |
| Anaerobutyricum soehngenii | 0.00482901104026025 | 0.0668642440284937 |
| Bacteroides thetaiotaomicron | 0.00601126372716994 | 0.0668642440284937 |
| Streptococcus parasanguinis_B | 0.00628745358385377 | 0.0668642440284937 |
| Fusicatenibacter saccharivorans | 0.00642423722270276 | 0.0668642440284937 |
| Bacteroides uniformis | 0.00737764641860981 | 0.0668642440284937 |
| Acetivibrio clariflavus | 0.00755361894540929 | 0.0668642440284937 |
| Prevotella copri_A | 0.00838874256956359 | 0.0668642440284937 |
| Lachnospira eligens_A | 0.0100969572394787 | 0.0668642440284937 |
| Clostridium sp001916075 | 0.0102625287393811 | 0.0668642440284937 |
| Phocaeicola dorei | 0.0106956016516212 | 0.0668642440284937 |
| Phocaeicola plebeius_A | 0.0111923290211677 | 0.0668642440284937 |
| Prevotella copri | 0.0121486952376346 | 0.0668642440284937 |
| Megamonas funiformis | 0.0122811876787029 | 0.0668642440284937 |
| Lachnospira rogosae_A | 0.0145622375992133 | 0.0751104886696266 |
| Blautia coccoides | 0.0211777302709256 | 0.0957339719981765 |
| Streptococcus salivarius | 0.0215905840957274 | 0.0957339719981765 |
| Dysosmobacter welbionis | 0.0223454075552701 | 0.0957339719981765 |
| Faecalibacillus intestinalis | 0.0224681771016129 | 0.0957339719981765 |
| Anaerobutyricum hallii | 0.0265363996142496 | 0.108356965091519 |
| Prevotella sp900290275 | 0.0293023586951307 | 0.114865246084912 |
| Dorea formicigenerans | 0.0311377836925146 | 0.117095095652783 |
| Lachnospira sp003537285 | 0.0350555058353093 | 0.117095095652783 |
| Blautia coccoides_A | 0.0353352225809545 | 0.117095095652783 |
| Collinsella sp003459245 | 0.0354854636824661 | 0.117095095652783 |
| Prevotella sp900557255 | 0.0358454374447294 | 0.117095095652783 |
| Blautia stercoris | 0.0383263018679181 | 0.121160567195354 |
| Blautia sp003287895 | 0.0451185058879085 | 0.13817542428172 |
| Enterocloster sp000431375 | 0.0475075862261892 | 0.138951890208769 |
| Blautia sp000432195 | 0.0485777322668554 | 0.138951890208769 |

Figure 4-H AHC vs. AMDD phylum Wilcoxon rank-sum test results at phylum and species levels for pairwise comparisons

| AHC-AMDD 门水平 | p.value | fdr |
| --- | --- | --- |
| Firmicutes | 0.0194250850213363 | 0.15540068017069 |

Figure 4-I AHC vs. AMDD species Wilcoxon rank-sum test results at phylum and species levels for pairwise comparisons

|  | p.value | fdr |
| --- | --- | --- |
| Prevotella stercorea | 0.014576378551445 | 0.521426239063459 |
| Schaedlerella sp900066545 | 0.0161433126531096 | 0.521426239063459 |
| Blautia hominis | 0.017003029534678 | 0.521426239063459 |
| Streptococcus salivarius | 0.0307183884304872 | 0.533145499248208 |
| Streptococcus thermophilus | 0.0333619658649241 | 0.533145499248208 |
| Ruthenibacterium lactatiformans | 0.0371098367660603 | 0.533145499248208 |
| Blautia sp000432195 | 0.0491583484673065 | 0.533145499248208 |

Figure 5.Correlation Heatmap between gut microbiota (species level) and clinical symptom scores.(A-D) TMDD vs. THC group: (A) heatmap of overall correlations; (B) HAMD subscales; (C) PSQI subscales; (D) SCL-90 subscales.(E-H) AMDD vs. AHC group: (E) heatmap of overall correlations; (F) HAMD subscales; (G) PSQI subscales; (H) SCL-90 subscales. Only correlations with P<0.05 are shown in heatmaps.

## Figure5-A TMDD vs. THC heatmap of overall correlation FDR矫正值

| 菌种 | HAMD_total | HAMA_total | SCL-90 Mean | Zung Anxiety | Zung Depression | PSQI Total | MoCA Total |
| --- | --- | --- | --- | --- | --- | --- | --- |
| Prevotella copri | 0.285 | 0.968 | 0.425 | 0.825 | 0.245 | 0.215 | 0.262 |
| Phocaeicola vulgatus | 0.510 | 0.285 | 0.825 | 0.968 | 0.042 | 0.355 | 0.385 |
| Bifidobacterium pseudocatenulatum | 0.345 | 0.190 | 0.850 | 0.462 | 0.925 | 0.775 | 0.510 |
| Phocaeicola plebeius_A | 0.465 | 0.495 | 0.565 | 0.138 | 0.465 | 0.955 | 0.840 |
| Fusicatenibacter saccharivorans | 0.810 | 0.720 | 0.535 | 0.990 | 0.605 | 0.990 | 0.465 |
| Agathobacter rectalis | 0.160 | 0.215 | 0.720 | 0.405 | 0.060 | 0.520 | 0.290 |
| Megamonas funiformis | 0.905 | 0.365 | 0.360 | 0.610 | 0.490 | 0.480 | 0.042 |
| Enterocloster sp000431375 | 0.660 | 0.955 | 0.675 | 0.082 | 0.465 | 0.490 | 0.640 |
| Rhodanobacter denitrificans | 0.190 | 0.730 | 0.370 | 0.395 | 0.745 | 0.500 | 0.570 |
| Agathobacter faecis | 0.375 | 0.045 | 0.955 | 0.445 | 0.680 | 0.980 | 0.260 |
| Pyrinomonas methylaliphatogenes | 0.350 | 0.700 | 0.320 | 0.260 | 0.680 | 0.700 | 0.640 |
| Bacteroides uniformis | 0.905 | 0.998 | 0.640 | 0.880 | 0.895 | 0.685 | 0.330 |
| Bifidobacterium longum | 0.730 | 0.955 | 0.415 | 0.820 | 0.790 | 0.740 | 0.500 |
| Bacteroides stercoris | 0.535 | 0.390 | 0.210 | 0.020 | 0.520 | 0.915 | 0.650 |
| Roseburia sp900552665 | 0.570 | 0.990 | 0.825 | 0.925 | 0.450 | 0.710 | 0.680 |

## Figure5-B:TMDD vs. THC grou HAMD subscales FDR矫正值

| 菌种 | HAMD_total | HAMD_severity | HAMD_anxiety | HAMD_weight | HAMD_cognition | HAMD_diurnal | HAMD_retardation | HAMD_sleep | HAMD_hopelessness |
| --- | --- | --- | --- | --- | --- | --- | --- | --- | --- |
| Prevotella copri | 0.262 | 0.362 | 0.392 | 0.105 | 0.298 | 0.140 | 0.065 | 0.405 | 0.200 |
| Phocaeicola vulgatus | 0.445 | 0.305 | 0.205 | 0.395 | 0.412 | 0.260 | 0.460 | 0.262 | 0.259 |
| Bifidobacterium pseudocatenulatum | 0.315 | 0.415 | 0.418 | 0.340 | 0.340 | 0.0245 | 0.351 | 0.135 | 0.488 |
| Phocaeicola plebeius_A | 0.390 | 0.425 | 0.440 | 0.238 | 0.115 | 0.298 | 0.472 | 0.488 | 0.490 |
| Fusicatenibacter saccharivorans | 0.630 | 0.385 | 0.210 | 0.310 | 0.395 | 0.490 | 0.162 | 0.490 | 0.495 |
| Agathobacter rectalis | 0.155 | 0.418 | 0.158 | 0.395 | 0.368 | 0.460 | 0.190 | 0.412 | 0.155 |
| Megamonas funiformis | 0.695 | 0.410 | 0.298 | 0.262 | 0.395 | 0.325 | 0.230 | 0.068 | 0.462 |
| Enterocloster sp000431375 | 0.532 | 0.286 | 0.410 | 0.395 | 0.205 | 0.108 | 0.210 | 0.455 | 0.388 |
| Rhodanobacter denitrificans | 0.148 | 0.395 | 0.115 | 0.490 | 0.208 | 0.395 | 0.425 | 0.384 | 0.085 |
| Agathobacter faecis | 0.345 | 0.390 | 0.458 | 0.395 | 0.258 | 0.192 | 0.395 | 0.192 | 0.412 |
| Pyrinomonas methylaliphatogenes | 0.320 | 0.395 | 0.138 | 0.438 | 0.426 | 0.359 | 0.395 | 0.248 | 0.165 |
| Bacteroides uniformis | 0.695 | 0.395 | 0.225 | 0.248 | 0.492 | 0.455 | 0.192 | 0.490 | 0.395 |
| Bifidobacterium longum | 0.570 | 0.379 | 0.190 | 0.490 | 0.465 | 0.490 | 0.238 | 0.490 | 0.245 |
| Bacteroides stercoris | 0.455 | 0.490 | 0.412 | 0.395 | 0.490 | 0.395 | 0.395 | 0.490 | 0.368 |
| Roseburia sp900552665 | 0.480 | 0.395 | 0.280 | 0.490 | 0.465 | 0.490 | 0.460 | 0.353 | 0.175 |

## Figure5-C: TMDD vs. THC PSQI subscales FDR矫正值

| 菌种 | Total | Quality | Latency | Duration | Efficiency | Disturbances | Medication | Daytime |
| --- | --- | --- | --- | --- | --- | --- | --- | --- |
| Prevotella copri | 0.215 | 0.425 | 0.485 | 0.825 | 0.215 | 0.485 | 0.485 | 0.485 |
| Phocaeicola vulgatus | 0.355 | 0.475 | 0.625 | 0.775 | 0.485 | 0.485 | 0.785 | 0.485 |
| Bifidobacterium pseudocatenulatum | 0.775 | 0.785 | 0.685 | 0.485 | 0.485 | 0.485 | 0.485 | 0.485 |
| Phocaeicola plebeius_A | 0.955 | 0.485 | 0.485 | 0.485 | 0.685 | 0.775 | 0.485 | 0.485 |
| Fusicatenibacter saccharivorans | 0.990 | 0.485 | 0.485 | 0.485 | 0.485 | 0.485 | 0.485 | 0.485 |
| Agathobacter rectalis | 0.520 | 0.425 | 0.485 | 0.485 | 0.485 | 0.425 | 0.485 | 0.485 |
| Megamonas funiformis | 0.480 | 0.485 | 0.485 | 0.485 | 0.485 | 0.485 | 0.485 | 0.485 |
| Enterocloster sp000431375 | 0.490 | 0.485 | 0.485 | 0.775 | 0.425 | 0.485 | 0.485 | 0.485 |
| Rhodanobacter denitrificans | 0.500 | 0.485 | 0.625 | 0.485 | 0.485 | 0.485 | 0.485 | 0.485 |
| Agathobacter faecis | 0.980 | 0.485 | 0.485 | 0.485 | 0.485 | 0.485 | 0.485 | 0.485 |
| Pyrinomonas methylaliphatogenes | 0.700 | 0.485 | 0.485 | 0.485 | 0.485 | 0.485 | 0.485 | 0.485 |
| Bacteroides uniformis | 0.685 | 0.485 | 0.485 | 0.485 | 0.485 | 0.485 | 0.485 | 0.485 |
| Bifidobacterium longum | 0.740 | 0.485 | 0.485 | 0.485 | 0.485 | 0.485 | 0.485 | 0.485 |
| Bacteroides stercoris | 0.915 | 0.485 | 0.485 | 0.485 | 0.485 | 0.485 | 0.485 | 0.485 |
| Roseburia sp900552665 | 0.710 | 0.485 | 0.485 | 0.485 | 0.485 | 0.485 | 0.485 | 0.485 |

## Figure5-D: TMDD vs. THC SCL-90 subscales FDR矫正值

| 菌种 | GSI | Somatization | Obsessive-Compulsive | Interpersonal Sensitivity | Depression | Anxiety | Hostility | Phobic Anxiety | Paranoid Ideation | Psychoticism | Additional |
| --- | --- | --- | --- | --- | --- | --- | --- | --- | --- | --- | --- |
| Prevotella copri | 0.425 | 0.138 | 0.462 | 0.298 | 0.515 | 0.366 | 0.560 | 0.445 | 0.582 | 0.245 | 0.362 |
| Phocaeicola vulgatus | 0.825 | 0.490 | 0.335 | 0.588 | 0.478 | 0.298 | 0.387 | 0.775 | 0.162 | 0.925 | 0.260 |
| Bifidobacterium pseudocatenulatum | 0.818 | 0.480 | 0.908 | 0.243 | 0.399 | 0.679 | 0.573 | 0.438 | 0.876 | 0.460 | 0.978 |
| Phocaeicola plebeius_A | 0.530 | 0.265 | 0.888 | 0.763 | 0.752 | 0.510 | 0.269 | 0.295 | 0.534 | 0.477 | 0.733 |
| Fusicatenibacter saccharivorans | 0.483 | 0.299 | 0.354 | 0.187 | 0.249 | 0.316 | 0.679 | 0.549 | 0.066 | 0.365 | 0.241 |
| Agathobacter rectalis | 0.650 | 0.437 | 0.575 | 0.701 | 0.467 | 0.902 | 0.280 | 0.696 | 0.826 | 0.585 | 0.284 |
| Megamonas funiformis | 0.278 | 0.498 | 0.581 | 0.874 | 0.121 | 0.325 | 0.623 | 0.437 | 0.578 | 0.158 | 0.035 |
| Enterocloster sp000431375 | 0.627 | 0.321 | 0.759 | 0.917 | 0.829 | 0.585 | 0.729 | 0.254 | 0.465 | 0.726 | 0.484 |
| Rhodanobacter denitrificans | 0.297 | 0.194 | 0.465 | 0.658 | 0.955 | 0.483 | 0.028 | 0.245 | 0.027 | 0.656 | 0.898 |
| Agathobacter faecis | 0.955 | 0.507 | 0.985 | 0.422 | 0.610 | 0.735 | 0.704 | 0.419 | 0.735 | 0.467 | 0.798 |
| Pyrinomonas methylaliphatogenes | 0.236 | 0.227 | 0.391 | 0.558 | 0.870 | 0.396 | 0.025 | 0.162 | 0.025 | 0.551 | 0.822 |
| Bacteroides uniformis | 0.575 | 0.293 | 0.339 | 0.266 | 0.313 | 0.433 | 0.469 | 0.390 | 0.094 | 0.415 | 0.256 |
| Bifidobacterium longum | 0.348 | 0.573 | 0.785 | 0.204 | 0.125 | 0.089 | 0.671 | 0.854 | 0.939 | 0.148 | 0.409 |
| Bacteroides stercoris | 0.141 | 0.086 | 0.525 | 0.136 | 0.658 | 0.107 | 0.709 | 0.107 | 0.160 | 0.280 | 0.020 |
| Roseburia sp900552665 | 0.767 | 0.679 | 0.312 | 0.569 | 0.497 | 0.271 | 0.251 | 0.457 | 0.172 | 0.932 | 0.377 |

Figure5-E: AMDD vs. AHC group: heatmap of overall correlations FDR矫正值

| 菌种 | HAMD_total | HAMA_total | SCL-90 Mean | Zung Anxiety | Zung Depression | PSQI Total | MoCA Total |
| --- | --- | --- | --- | --- | --- | --- | --- |
| Prevotella copri | 0.285 | 0.968 | 0.425 | 0.825 | 0.245 | 0.215 | 0.262 |
| Phocaeicola vulgatus | 0.510 | 0.285 | 0.825 | 0.968 | 0.042 | 0.355 | 0.385 |
| Bifidobacterium pseudocatenulatum | 0.345 | 0.190 | 0.850 | 0.462 | 0.925 | 0.775 | 0.510 |
| Phocaeicola plebeius_A | 0.465 | 0.495 | 0.565 | 0.138 | 0.465 | 0.955 | 0.840 |
| Fusicatenibacter saccharivorans | 0.810 | 0.720 | 0.535 | 0.990 | 0.605 | 0.990 | 0.465 |
| Agathobacter rectalis | 0.160 | 0.215 | 0.720 | 0.405 | 0.060 | 0.520 | 0.290 |
| Megamonas funiformis | 0.905 | 0.365 | 0.360 | 0.610 | 0.490 | 0.480 | 0.042 |
| Enterocloster sp000431375 | 0.660 | 0.955 | 0.675 | 0.082 | 0.465 | 0.490 | 0.640 |
| Rhodanobacter denitrificans | 0.190 | 0.730 | 0.370 | 0.395 | 0.745 | 0.500 | 0.570 |
| Agathobacter faecis | 0.375 | 0.045 | 0.955 | 0.445 | 0.680 | 0.980 | 0.260 |
| Pyrinomonas methylaliphatogenes | 0.350 | 0.700 | 0.320 | 0.260 | 0.680 | 0.700 | 0.640 |
| Bacteroides uniformis | 0.905 | 0.998 | 0.640 | 0.880 | 0.895 | 0.685 | 0.330 |
| Bifidobacterium longum | 0.730 | 0.955 | 0.415 | 0.820 | 0.790 | 0.740 | 0.500 |
| Bacteroides stercoris | 0.535 | 0.390 | 0.210 | 0.020 | 0.520 | 0.915 | 0.650 |
| Roseburia sp900552665 | 0.570 | 0.990 | 0.825 | 0.925 | 0.450 | 0.710 | 0.680 |

Figure5-F: AMDD vs. AHC group: HAMD subscales FDR矫正值

| 菌种 | HAMD_total | HAMD_severity | HAMD_anxiety | HAMD_weight | HAMD_cognition | HAMD_diurnal | HAMD_retardation | HAMD_sleep | HAMD_hopelessness |
| --- | --- | --- | --- | --- | --- | --- | --- | --- | --- |
| Bacteroides stercoris | 0.370 | 0.890 | 0.486 | 0.802 | 0.043 | 0.612 | 0.305 | 0.486 | 0.860 |
| Bifidobacterium longum | 0.765 | 0.968 | 0.930 | 0.920 | 0.765 | 0.968 | 0.968 | 0.950 | 0.950 |
| Phocaeicola vulgatus | 0.275 | 0.575 | 0.118 | 0.860 | 0.389 | 0.930 | 0.092 | 0.596 | 0.890 |
| Agathobacter rectalis | 0.265 | 0.585 | 0.328 | 0.305 | 0.049 | 0.378 | 0.082 | 0.456 | 0.968 |
| Faecalibacterium prausnitzii_C | 0.775 | 0.782 | 0.765 | 0.950 | 0.930 | 0.055 | 0.828 | 0.860 | 0.930 |
| Agathobacter faecis | 0.275 | 0.950 | 0.486 | 0.310 | 0.285 | 0.408 | 0.275 | 0.040 | 0.968 |
| Gemmiger qucibialis | 0.370 | 0.875 | 0.486 | 0.436 | 0.536 | 0.535 | 0.416 | 0.782 | 0.930 |
| Anaerobutyricum hallii | 0.968 | 0.968 | 0.930 | 0.890 | 0.968 | 0.920 | 0.968 | 0.968 | 0.950 |
| Prevotella copri | 0.822 | 0.668 | 0.968 | 0.428 | 0.968 | 0.968 | 0.555 | 0.930 | 0.968 |
| Megamonas funiformis | 0.718 | 0.968 | 0.765 | 0.370 | 0.536 | 0.596 | 0.536 | 0.740 | 0.968 |
| Enterocloster sp000431375 | 0.622 | 0.968 | 0.575 | 0.315 | 0.828 | 0.436 | 0.555 | 0.648 | 0.968 |
| Rhodanobacter sp001617365 | 0.740 | 0.992 | 0.920 | 0.860 | 0.860 | 0.782 | 0.660 | 0.782 | 0.968 |
| Bifidobacterium pseudocatenulatum | 0.925 | 0.968 | 0.825 | 0.815 | 0.390 | 0.486 | 0.992 | 0.610 | 0.968 |
| Bacteroides uniformis | 0.370 | 0.340 | 0.486 | 0.968 | 0.370 | 0.436 | 0.285 | 0.536 | 0.890 |
| Phocaeicola plebeius_A | 0.612 | 0.968 | 0.555 | 0.688 | 0.968 | 0.715 | 0.555 | 0.688 | 0.968 |

Figure5-G: AMDD vs. AHC group: PSQI subscales FDR矫正值

| 菌种 | Quality | Latency | Duration | Efficiency | Disturbances | Medication | Daytime |
| --- | --- | --- | --- | --- | --- | --- | --- |
| Bacteroides stercoris | 0.925 | 0.968 | 0.500 | 0.438 | 0.890 | 0.518 | 0.002 |
| Bifidobacterium longum | 0.610 | 0.680 | 0.438 | 0.438 | 0.968 | 0.775 | 0.968 |
| Phocaeicola vulgatus | 0.968 | 0.668 | 0.518 | 0.930 | 0.738 | 0.380 | 0.998 |
| Agathobacter rectalis | 0.998 | 0.998 | 0.890 | 0.438 | 0.438 | 0.998 | 0.438 |
| Faecalibacterium prausnitzii_C | 0.480 | 0.380 | 0.968 | 0.860 | 0.930 | 0.930 | 0.930 |
| Agathobacter faecis | 0.310 | 0.890 | 0.680 | 0.668 | 0.860 | 0.338 | 0.620 |
| Gemmiger qucibialis | 0.380 | 0.338 | 0.668 | 0.180 | 0.488 | 0.775 | 0.438 |
| Anaerobutyricum hallii | 0.738 | 0.560 | 0.930 | 0.668 | 0.968 | 0.775 | 0.968 |
| Prevotella copri | 0.968 | 0.538 | 0.998 | 0.790 | 0.930 | 0.610 | 0.968 |
| Megamonas funiformis | 0.500 | 0.968 | 0.800 | 0.610 | 0.968 | 0.860 | 0.738 |
| Enterocloster sp000431375 | 0.998 | 0.680 | 0.968 | 0.488 | 0.968 | 0.380 | 0.968 |
| Rhodanobacter sp001617365 | 0.180 | 0.338 | 0.860 | 0.890 | 0.738 | 0.930 | 0.518 |
| Bifidobacterium pseudocatenulatum | 0.610 | 0.438 | 0.310 | 0.890 | 0.968 | 0.438 | 0.780 |
| Bacteroides uniformis | 0.890 | 0.930 | 0.738 | 0.998 | 0.338 | 0.775 | 0.998 |
| Phocaeicola plebeius_A | 0.468 | 0.800 | 0.998 | 0.998 | 0.998 | 0.380 | 0.790 |

Figure5-H: AMDD vs. AHC group: SCL-90 subscales FDR矫正值

| 菌种 | GSI | Somatization | Obsessive-Compulsive | Interpersonal Sensitivity | Depression | Anxiety | Hostility | Phobic Anxiety | Paranoid Ideation | Psychoticism | Additional |
| --- | --- | --- | --- | --- | --- | --- | --- | --- | --- | --- | --- |
| Bacteroides stercoris | 0.825 | 0.860 | 0.930 | 0.790 | 0.740 | 0.430 | 0.968 | 0.968 | 0.968 | 0.860 | 0.820 |
| Bifidobacterium longum | 0.530 | 0.990 | 0.148 | 0.155 | 0.340 | 0.148 | 0.560 | 0.340 | 0.860 | 0.340 | 0.620 |
| Phocaeicola vulgatus | 0.775 | 0.740 | 0.890 | 0.790 | 0.620 | 0.540 | 0.340 | 0.998 | 0.088 | 0.720 | 0.720 |
| Agathobacter rectalis | 0.968 | 0.055 | 0.428 | 0.860 | 0.620 | 0.860 | 0.968 | 0.968 | 0.680 | 0.608 | 0.340 |
| Faecalibacterium prausnitzii_C | 0.860 | 0.068 | 0.930 | 0.890 | 0.860 | 0.890 | 0.968 | 0.560 | 0.718 | 0.620 | 0.540 |
| Agathobacter faecis | 0.990 | 0.185 | 0.968 | 0.340 | 0.968 | 0.968 | 0.968 | 0.860 | 0.860 | 0.620 | 0.480 |
| Gemmiger qucibialis | 0.740 | 0.300 | 0.890 | 0.438 | 0.790 | 0.968 | 0.998 | 0.968 | 0.718 | 0.740 | 0.680 |
| Anaerobutyricum hallii | 0.740 | 0.620 | 0.238 | 0.540 | 0.238 | 0.340 | 0.620 | 0.820 | 0.890 | 0.430 | 0.620 |
| Prevotella copri | 0.968 | 0.968 | 0.930 | 0.968 | 0.890 | 0.680 | 0.680 | 0.890 | 0.148 | 0.890 | 0.380 |
| Megamonas funiformis | 0.860 | 0.082 | 0.165 | 0.340 | 0.198 | 0.340 | 0.380 | 0.380 | 0.430 | 0.095 | 0.340 |
| Enterocloster sp000431375 | 0.990 | 0.720 | 0.380 | 0.860 | 0.790 | 0.438 | 0.340 | 0.780 | 0.238 | 0.890 | 0.890 |
| Rhodanobacter sp001617365 | 0.990 | 0.280 | 0.118 | 0.655 | 0.340 | 0.620 | 0.660 | 0.620 | 0.340 | 0.340 | 0.340 |
| Bifidobacterium pseudocatenulatum | 0.740 | 0.340 | 0.340 | 0.340 | 0.165 | 0.680 | 0.968 | 0.408 | 0.890 | 0.890 | 0.340 |
| Bacteroides uniformis | 0.860 | 0.998 | 0.930 | 0.340 | 0.790 | 0.740 | 0.890 | 0.055 | 0.340 | 0.340 | 0.968 |
| Phocaeicola plebeius_A | 0.620 | 0.080 | 0.740 | 0.340 | 0.990 | 0.890 | 0.620 | 0.860 | 0.790 | 0.968 | 0.340 |

Figure 6. Plasma metabolic profiles and pathway alterations in adolescent and adult MDD patients compared to age-matched healthy controls.(H-J) KEGG pathway enrichment analysis bubble plots:(H) AHC vs. THC;(I) THC vs. TMDD;(J) AHC vs. AMDD.

## 图6-H :KEGG pathway enrichment analysis bubble plots: THC-AHC值and FDR值

| layer3 | pvalue | FDR |
| --- | --- | --- |
| Glutathione metabolism | 0.009339158 | 0.999433913 |
| Secondary bile acid biosynthesis | 0.023077186 | 0.999433913 |
| C5-Branched dibasic acid metabolism | 0.023077186 | 0.999433913 |
| Ascorbate and aldarate metabolism | 0.026234301 | 0.999433913 |
| Arginine and proline metabolism | 0.026513439 | 0.859035427 |
| Pathways of neurodegeneration - multiple diseases | 0.043964779 | 0.999433913 |
| Bile secretion | 0.049405127 | 0.999433913 |
| Arginine biosynthesis | 0.109402175 | 0.999433913 |
| Primary bile acid biosynthesis | 0.120672391 | 0.999433913 |
| Phospholipase D signaling pathway | 0.124756525 | 0.999433913 |
| Amyotrophic lateral sclerosis | 0.124756525 | 0.999433913 |
| Proximal tubule bicarbonate reclamation | 0.129449822 | 0.999433913 |
| Taurine and hypotaurine metabolism | 0.129449822 | 0.999433913 |
| Arachidonic acid metabolism | 0.129449822 | 0.999433913 |
| Neuroactive ligand-receptor interaction | 0.219402283 | 0.999433913 |
| Pentose and glucuronate interconversions | 0.240211173 | 0.999433913 |
| Citrate cycle (TCA cycle) | 0.240211173 | 0.999433913 |
| Carbon fixation pathways in prokaryotes | 0.240211173 | 0.999433913 |
| Butanoate metabolism | 0.286340018 | 0.999433913 |
| Phosphotransferase system (PTS) | 0.286340018 | 0.999433913 |
| Oxytocin signaling pathway | 0.286340018 | 0.999433913 |
| GABAergic synapse | 0.286340018 | 0.999433913 |
| Metabolism of xenobiotics by cytochrome P450 | 0.286340018 | 0.999433913 |
| Phenazine biosynthesis | 0.286340018 | 0.999433913 |
| Choline metabolism in cancer | 0.286340018 | 0.999433913 |
| Amoebiasis | 0.286340018 | 0.999433913 |
| Betalain biosynthesis | 0.286340018 | 0.999433913 |
| D-Amino acid metabolism | 0.292530783 | 0.999433913 |
| Biosynthesis of terpenoids and steroids | 0.30060964 | 0.999433913 |
| Biosynthesis of alkaloids derived from ornithine, lysine and nicotinic acid | 0.321614148 | 0.999433913 |

## 图6-I :KEGG pathway enrichment analysis bubble plots: THC-TMDD P值and FDR值

| layer3 | pvalue | FDR |
| --- | --- | --- |
| Ascorbate and aldarate metabolism | 0.006810752 | 0.998936863 |
| Glutathione metabolism | 0.015639382 | 0.998936863 |
| Pathways of neurodegeneration - multiple diseases | 0.058074988 | 0.998936863 |
| Butanoate metabolism | 0.058074988 | 0.998936863 |
| Pentose and glucuronate interconversions | 0.077546037 | 0.998936863 |
| Arginine and proline metabolism | 0.136322267 | 0.998936863 |
| Sulfur metabolism | 0.150160064 | 0.998936863 |
| Amyotrophic lateral sclerosis | 0.150160064 | 0.998936863 |
| Biosynthesis of terpenoids and steroids | 0.154530638 | 0.998936863 |
| Arginine biosynthesis | 0.154530638 | 0.998936863 |
| ABC transporters | 0.155757025 | 0.998936863 |
| C5-Branched dibasic acid metabolism | 0.162137689 | 0.998936863 |
| cAMP signaling pathway | 0.165005203 | 0.998936863 |
| Proximal tubule bicarbonate reclamation | 0.165005203 | 0.998936863 |
| Taurine and hypotaurine metabolism | 0.165005203 | 0.998936863 |
| D-Amino acid metabolism | 0.200425137 | 0.998936863 |
| Biosynthesis of various antibiotics | 0.240774279 | 0.998936863 |
| Carbon metabolism | 0.240774279 | 0.998936863 |
| Biosynthesis of alkaloids derived from ornithine, lysine and nicotinic acid | 0.248862331 | 0.998936863 |
| Caprolactam degradation | 0.265951563 | 0.998936863 |
| Lysine biosynthesis | 0.265951563 | 0.998936863 |
| Pantothenate and CoA biosynthesis | 0.296193952 | 0.998936863 |
| Carbon fixation pathways in prokaryotes | 0.296193952 | 0.998936863 |
| Citrate cycle (TCA cycle) | 0.296193952 | 0.998936863 |
| Bile secretion | 0.329988436 | 0.998936863 |
| Amoebiasis | 0.334330216 | 0.998936863 |
| Choline metabolism in cancer | 0.334330216 | 0.998936863 |
| Metabolism of xenobiotics by cytochrome P450 | 0.334330216 | 0.998936863 |
| Betalain biosynthesis | 0.334330216 | 0.998936863 |
| Quorum sensing | 0.334330216 | 0.998936863 |

## 图6-J KEGG pathway enrichment analysis bubble plots: AHC-AMDD P值and FDR值

| layer3 | pvalue | FDR |
| --- | --- | --- |
| Two-component system | 0.018850561 | 0.999781592 |
| Purine metabolism | 0.028930248 | 0.999781592 |
| Phenazine biosynthesis | 0.050980296 | 0.999781592 |
| Carbon fixation pathways in prokaryotes | 0.066411024 | 0.999781592 |
| Citrate cycle (TCA cycle) | 0.066411024 | 0.999781592 |
| Glyoxylate and dicarboxylate metabolism | 0.069992109 | 0.999781592 |
| Alanine, aspartate and glutamate metabolism | 0.069992109 | 0.999781592 |
| Carbon fixation in photosynthetic organisms | 0.137680711 | 0.999781592 |
| Nitrogen metabolism | 0.137680711 | 0.999781592 |
| Glutamatergic synapse | 0.137680711 | 0.999781592 |
| Amyotrophic lateral sclerosis | 0.137680711 | 0.999781592 |
| Stilbenoid, diarylheptanoid and gingerol biosynthesis | 0.137680711 | 0.999781592 |
| Fatty acid degradation | 0.137680711 | 0.999781592 |
| Biosynthesis of siderophore group nonribosomal peptides | 0.1473695 | 0.999781592 |
| Taste transduction | 0.1473695 | 0.999781592 |
| Glucagon signaling pathway | 0.1473695 | 0.999781592 |
| Proximal tubule bicarbonate reclamation | 0.1473695 | 0.999781592 |
| Biosynthesis of phenylpropanoids | 0.184999953 | 0.999781592 |
| Carbon metabolism | 0.208868411 | 0.999781592 |
| alpha-Linolenic acid metabolism | 0.268807215 | 0.999781592 |
| Fatty acid biosynthesis | 0.268807215 | 0.999781592 |
| Ferroptosis | 0.268807215 | 0.999781592 |
| Central carbon metabolism in cancer | 0.303126625 | 0.999781592 |
| Choline metabolism in cancer | 0.311081541 | 0.999781592 |
| GABAergic synapse | 0.311081541 | 0.999781592 |
| Butanoate metabolism | 0.311081541 | 0.999781592 |
| Vitamin B6 metabolism | 0.311081541 | 0.999781592 |
| Pathways of neurodegeneration - multiple diseases | 0.311081541 | 0.999781592 |
| Pyrimidine metabolism | 0.311081541 | 0.999781592 |
| Nucleotide metabolism | 0.311081541 | 0.999781592 |

Figure 7.Correlations between plasma metabolites and clinical symptom scores.(A-D) TMDD vs. THC group: (A) heatmap of overall correlations; (B) HAMD subscales; (C) PSQI subscales，矫正FDR均大于0.05; (D) SCL-90 subscales，矫正FDR均大于0.05.(E-H) AMDD vs. AHC group: (E) heatmap of overall correlations; (F) HAMD subscales; (G) PSQI subscales; (H) SCL-90

## 图7-A heatmap of overall correlations FDR 校正后 q 值矩阵

| 代谢物 | HAMD_total | HAMA_total | SCL-90 Mean | Zung Anxiety | Zung Depression | PSQI Total | MoCA |
| --- | --- | --- | --- | --- | --- | --- | --- |
| (E,E)-2,9,9-Trimethyl-... | 0.813 | 0.695 | 0.956 | 0.648 | 0.787 | 0.879 | 0.725 |
| Aminocaproic acid | 0.925 | 0.648 | 0.757 | 0.608 | 0.480 | 0.968 | 0.891 |
| Gibbilimbol C | 0.825 | 0.891 | 0.742 | 0.968 | 0.891 | 0.879 | 0.956 |
| 2-heptyl-3-hydroxy-4-quinolone | 0.956 | 0.813 | 0.891 | 0.825 | 0.608 | 0.960 | 0.695 |
| Palmitic amide | 0.215 | 0.275 | 0.104 | 0.393 | 0.445 | 0.160 | 0.891 |
| Geranylacetone | 0.775 | 0.925 | 0.471 | 0.925 | 0.813 | 0.879 | 0.891 |
| 3-Hydroxybutyric acid | 0.648 | 0.891 | 0.510 | 0.891 | 0.925 | 0.799 | 0.725 |
| (S)-(+)-alpha-Phellandrene | 0.464 | 0.352 | 0.414 | 0.335 | 0.386 | 0.638 | 0.813 |
| N-Methyl-calystegine B2 | 0.295 | 0.480 | 0.408 | 0.352 | 0.408 | 0.500 | 0.608 |
| 5-oxoproline | 0.725 | 0.968 | 0.561 | 0.891 | 0.925 | 0.879 | 0.675 |

## 图7-B HAMD subscale FDR 校正后 q 值矩阵

| 代谢物 | HAMD_total | HAMD_anxiety | HAMD_weight | HAMD_cognition | HAMD_diurnal | HAMD_retardation | HAMD_sleep | HAMD_hopelessness |
| --- | --- | --- | --- | --- | --- | --- | --- | --- |
| (E,E)-2,9,9-Trimethyl-... | 0.813 | 0.695 | 0.738 | 0.891 | 0.787 | 0.925 | 0.956 | 0.825 |
| Aminocaproic acid | 0.925 | 0.813 | 0.695 | 0.968 | 0.903 | 0.925 | 0.875 | 0.984 |
| Gibbilimbol C | 0.825 | 0.891 | 0.851 | 0.738 | 0.725 | 0.825 | 0.903 | 0.984 |
| 2-heptyl-3-hydroxy-4-quinolone | 0.956 | 0.891 | 0.984 | 0.968 | 0.925 | 0.925 | 0.956 | 0.956 |
| Palmitic amide | 0.215 | 0.348 | 0.416 | 0.185 | 0.393 | 0.425 | 0.545 | 0.408 |
| Geranylacetone | 0.775 | 0.825 | 0.738 | 0.725 | 0.903 | 0.925 | 0.787 | 0.851 |
| 3-Hydroxybutyric acid | 0.648 | 0.738 | 0.968 | 0.480 | 0.573 | 0.695 | 0.787 | 0.825 |
| (S)-(+)-alpha-Phellandrene | 0.464 | 0.338 | 0.555 | 0.608 | 0.787 | 0.675 | 0.648 | 0.648 |
| N-Methyl-calystegine B2 | 0.295 | 0.438 | 0.531 | 0.445 | 0.338 | 0.348 | 0.555 | 0.438 |
| 5-oxoproline | 0.725 | 0.891 | 0.825 | 0.608 | 0.480 | 0.813 | 0.891 | 0.787 |

## 图7-C PSQI subscale

| 代谢物 | Total | Quality | Latency | Duration | Efficiency | Disturbances | Medication | Daytime |
| --- | --- | --- | --- | --- | --- | --- | --- | --- |
| (E,E)-2,9,9-Trimethyl-... | 0.879 | 0.912 | 0.839 | 0.825 | 0.710 | 0.638 | 0.648 | 0.684 |
| Aminocaproic acid | 0.968 | 0.854 | 0.918 | 0.984 | 0.877 | 0.552 | 0.275 | 0.784 |
| Gibbilimbol C | 0.879 | 0.930 | 0.984 | 0.858 | 0.863 | 0.960 | 0.852 | 0.813 |
| 2-heptyl-3-hydroxy-4-quinolone | 0.960 | 0.897 | 0.992 | 0.930 | 0.810 | 0.744 | 0.930 | 0.774 |
| Palmitic amide | 0.160 | 0.332 | 0.416 | 0.024 | 0.984 | 0.451 | 0.930 | 0.440 |
| Geranylacetone | 0.879 | 0.798 | 0.670 | 0.984 | 0.810 | 0.960 | 0.930 | 0.930 |
| 3-Hydroxybutyric acid | 0.799 | 0.897 | 0.984 | 0.681 | 0.684 | 0.609 | 0.879 | 0.813 |
| (S)-(+)-alpha-Phellandrene | 0.638 | 0.420 | 0.839 | 0.733 | 0.960 | 0.638 | 0.810 | 0.356 |
| N-Methyl-calystegine B2 | 0.500 | 0.528 | 0.366 | 0.761 | 0.774 | 0.813 | 0.984 | 0.366 |
| 5-oxoproline | 0.879 | 0.854 | 0.761 | 0.868 | 0.565 | 0.648 | 0.879 | 0.789 |

## 图7-D： TMDD与THC组SCL-90与代谢物相关性系数 矫正FDR的q值

| 代谢物 | 总分均分 | 躯体化 | 强迫 | 人际 | 抑郁 | 焦虑 | 敌对 | 恐怖 | 偏执 | 精神病性 | 其他 |
| --- | --- | --- | --- | --- | --- | --- | --- | --- | --- | --- | --- |
| (E,E)-2,9,9-Trimethyl-... | 0.925 | 0.892 | 0.925 | 0.925 | 0.903 | 0.968 | 0.925 | 0.757 | 0.875 | 0.925 | 0.955 |
| Aminocaproic acid | 0.757 | 0.713 | 0.851 | 0.825 | 0.811 | 0.695 | 0.738 | 0.925 | 0.738 | 0.757 | 0.811 |
| Gibbilimbol C | 0.738 | 0.738 | 0.771 | 0.793 | 0.834 | 0.842 | 0.648 | 0.675 | 0.811 | 0.925 | 0.687 |
| 2-heptyl-3-hydroxy-4-quinolone | 0.851 | 0.903 | 0.875 | 0.851 | 0.834 | 0.757 | 0.903 | 0.968 | 0.813 | 0.925 | 0.968 |
| Palmitic amide | 0.104 | 0.11 | 0.189 | 0.139 | 0.121 | 0.13 | 0.229 | 0.352 | 0.158 | 0.173 | 0.114 |
| Geranylacetone | 0.471 | 0.51 | 0.471 | 0.452 | 0.51 | 0.46 | 0.423 | 0.531 | 0.561 | 0.51 | 0.488 |
| 3-Hydroxybutyric acid | 0.51 | 0.484 | 0.55 | 0.531 | 0.573 | 0.617 | 0.348 | 0.648 | 0.648 | 0.793 | 0.471 |
| (S)-(+)-alpha-Phellandrene | 0.414 | 0.386 | 0.451 | 0.356 | 0.366 | 0.46 | 0.31 | 0.561 | 0.489 | 0.51 | 0.313 |
| N-Methyl-calystegine B2 | 0.408 | 0.536 | 0.356 | 0.536 | 0.386 | 0.364 | 0.675 | 0.968 | 0.445 | 0.414 | 0.334 |
| 5-oxoproline | 0.561 | 0.617 | 0.598 | 0.55 | 0.573 | 0.531 | 0.423 | 0.738 | 0.793 | 0.675 | 0.55 |

## 图7-E:AHC-AMDD heatmap of overall correlations;

| 代谢物 | HAMD_total | HAMA_total | SCL-90 Mean | Zung Anxiety | Zung Depression | PSQI Total | MoCA |
| --- | --- | --- | --- | --- | --- | --- | --- |
| Gibbilimbol C | 0.956 | 0.813 | 0.851 | 0.851 | 0.968 | 0.738 | 0.555 |
| Phthalic acid mono-2-ethylhexyl ester | 0.738 | 0.851 | 0.925 | 0.891 | 0.851 | 0.787 | 0.545 |
| **4-Acetylimidazo[4,5-c]pyridine** | 0.348 | **0.445** | **0.160** | **0.305** | **0.285** | 0.416 | **0.675** |
| Ammelide | 0.445 | 0.555 | 0.825 | 0.851 | 0.695 | 0.738 | 0.787 |
| L-Citramalic acid | 0.984 | 0.968 | 0.925 | 0.891 | 0.925 | 0.787 | 0.813 |
| Flucytosine | 0.464 | 0.510 | 0.648 | 0.787 | 0.555 | 0.738 | 0.608 |
| 9,10,13-TriHOME | 0.984 | 0.813 | 0.851 | 0.891 | 0.825 | 0.925 | 0.825 |
| Temozolomide | 0.648 | 0.695 | 0.956 | 0.851 | 0.555 | 0.648 | 0.510 |
| L-SelenoMethionine | 0.648 | 0.648 | 0.695 | 0.695 | 0.695 | 0.695 | 0.555 |
| 2-Amino-1,3,4-thiadiazole | 0.695 | 0.695 | 0.813 | 0.851 | 0.695 | 0.738 | 0.573 |

## 图7-F:AHC-AMDD HAMD subscales FDR 矫正q值

| 代谢物 | HAMD总分 | 焦虑因子 | 体重因子 | 认知障碍 | 日夜变化 | 迟缓因子 | 睡眠障碍 | 绝望感 |
| --- | --- | --- | --- | --- | --- | --- | --- | --- |
| Gibbilimbol C | 0.968 | 0.903 | 0.956 | 0.903 | 0.787 | 0.851 | 0.738 | 0.956 |
| Phthalic acid mono-2-ethylhexyl ester | 0.738 | 0.738 | 0.787 | 0.813 | 0.968 | 0.787 | 0.648 | 0.891 |
| 4-Acetylimidazo[4,5-c]pyridine | 0.545 | 0.531 | 0.393 | 0.695 | 0.984 | 0.738 | 0.738 | 0.695 |
| Ammelide | 0.531 | 0.648 | 0.445 | 0.608 | 0.891 | 0.738 | 0.903 | 0.608 |
| L-Citramalic acid | 0.968 | 0.968 | 0.984 | 0.813 | 0.903 | 0.984 | 0.968 | 0.925 |
| Flucytosine | 0.545 | 0.648 | 0.416 | 0.695 | 0.891 | 0.738 | 0.903 | 0.555 |
| 9,10,13-TriHOME | 0.813 | 0.825 | 0.851 | 0.925 | 0.903 | 0.825 | 0.695 | 0.968 |
| Temozolomide | 0.608 | 0.608 | 0.510 | 0.675 | 0.851 | 0.813 | 0.851 | 0.695 |
| L-SelenoMethionine | 0.648 | 0.695 | 0.480 | 0.738 | 0.813 | 0.738 | 0.813 | 0.648 |
| 2-Amino-1,3,4-thiadiazole | 0.608 | 0.695 | 0.445 | 0.695 | 0.851 | 0.787 | 0.787 | 0.648 |

## 图7-G：AHC-AMDD PSQI subscales FDR 矫正q值

| 代谢物 | Total | Quality | Latency | Duration | Efficiency | Disturbances | Medication | Daytime |
| --- | --- | --- | --- | --- | --- | --- | --- | --- |
| Gibbilimbol C | 0.738 | 0.851 | 0.648 | 0.925 | 0.675 | 0.984 | 0.738 | 0.738 |
| Phthalic acid mono-2-ethylhexyl ester | 0.787 | 0.695 | 0.956 | 0.555 | 0.825 | 0.925 | 0.648 | 0.825 |
| 4-Acetylimidazo[4,5-c]pyridine | 0.416 | 0.416 | 0.555 | 0.695 | 0.675 | 0.738 | 0.352 | 0.608 |
| Ammelide | 0.738 | 0.738 | 0.787 | 0.738 | 0.925 | 0.891 | 0.787 | 0.695 |
| L-Citramalic acid | 0.787 | 0.787 | 0.787 | 0.891 | 0.956 | 0.813 | 0.851 | 0.825 |
| Flucytosine | 0.738 | 0.695 | 0.813 | 0.787 | 0.903 | 0.813 | 0.738 | 0.695 |
| 9,10,13-TriHOME | 0.925 | 0.903 | 0.956 | 0.738 | 0.925 | 0.925 | 0.825 | 0.903 |
| Temozolomide | 0.648 | 0.608 | 0.738 | 0.648 | 0.825 | 0.825 | 0.648 | 0.675 |
| L-SelenoMethionine | 0.695 | 0.695 | 0.738 | 0.813 | 0.738 | 0.813 | 0.695 | 0.675 |
| 2-Amino-1,3,4-thiadiazole | 0.738 | 0.738 | 0.813 | 0.787 | 0.851 | 0.851 | 0.738 | 0.695 |

## 图7-H: AHC-AMDD SCL-90 FDR 矫正q值

| 代谢物 | GSI | Somatization | Obsessive-Compulsive | Interpersonal Sensitivity | Depression | Anxiety | Hostility | Phobic Anxiety | Paranoid Ideation | Psychoticism | Additional |
| --- | --- | --- | --- | --- | --- | --- | --- | --- | --- | --- | --- |
| Gibbilimbol C | 0.851 | 0.738 | 0.925 | 0.825 | 0.968 | 0.903 | 0.648 | 0.787 | 0.851 | 0.925 | 0.968 |
| Phthalic acid mono-2-ethylhexyl ester | 0.925 | 0.968 | 0.787 | 0.925 | 0.925 | 0.787 | 0.968 | 0.984 | 0.925 | 0.903 | 0.968 |
| 4-Acetylimidazo[4,5-c]pyridine | 0.160 | 0.104 | 0.189 | 0.160 | 0.121 | 0.087 | 0.189 | 0.305 | 0.130 | 0.264 | 0.155 |
| Ammelide | 0.825 | 0.813 | 0.825 | 0.813 | 0.891 | 0.738 | 0.851 | 0.903 | 0.851 | 0.825 | 0.787 |
| L-Citramalic acid | 0.925 | 0.851 | 0.968 | 0.925 | 0.968 | 0.851 | 0.925 | 0.968 | 0.851 | 0.925 | 0.891 |
| Flucytosine | 0.648 | 0.555 | 0.738 | 0.648 | 0.738 | 0.545 | 0.787 | 0.891 | 0.738 | 0.695 | 0.555 |
| 9,10,13-TriHOME | 0.851 | 0.851 | 0.968 | 0.825 | 0.851 | 0.891 | 0.787 | 0.813 | 0.851 | 0.851 | 0.903 |
| Temozolomide | 0.956 | 0.968 | 0.956 | 0.903 | 0.956 | 0.984 | 0.825 | 0.968 | 0.925 | 0.925 | 0.925 |
| L-SelenoMethionine | 0.695 | 0.695 | 0.738 | 0.675 | 0.787 | 0.555 | 0.825 | 0.825 | 0.738 | 0.813 | 0.648 |
| 2-Amino-1,3,4-thiadiazole | 0.813 | 0.813 | 0.787 | 0.813 | 0.891 | 0.695 | 0.851 | 0.925 | 0.825 | 0.825 | 0.787 |

Figure 9. Age-specific microbial–metabolite correlation networks in health and depression.(A)Correlation heatmap between top 20 metabolites and gut microbiota in THC vs. AHC（Mantel_p=0.341,Proc_p=0.08);(B)Correlation heatmap in THC vs. TMDD（Mantel_p=0.555 ,Proc_p=0.664); (C)Correlation heatmap in AHC vs. AMDD（Mantel_p=0.182，Proc_p=0.672).Only correlations with ∣r∣>0.5 and nominal P<0.05 are shown. Red indicates positive correlation, blue indicates negative correlation. The intensity of color represents the magnitude of the correlation coefficient.
